# Cell-Type–Specific Transcriptional Profiles of the Dimorphic Pathogen *Penicillium marneffei* Reflect Distinct Reproductive, Morphological, and Environmental Demands

**DOI:** 10.1534/g3.113.006809

**Published:** 2013-11-01

**Authors:** Shivani Pasricha, Michael Payne, David Canovas, Luke Pase, Nathamon Ngaosuwankul, Sally Beard, Alicia Oshlack, Gordon K. Smyth, Sansanee C. Chaiyaroj, Kylie J. Boyce, Alex Andrianopoulos

**Affiliations:** * Department of Genetics, University of Melbourne, Victoria 3010, Australia; † Department of Microbiology, Faculty of Science, Mahidol University, Bangkok 10400, Thailand; ‡ Bioinformatics Division, Walter and Eliza Hall Institute of Medical Research, Parkville, Victoria 3052, Australia; § Department of Mathematics and Statistics, University of Melbourne, Victoria 3010, Australia

**Keywords:** *Penicillium marneffei*, *Talaromyces marneffei*, microarray, hyphae, conidiation, yeast, dimorphic switch, cell wall

## Abstract

*Penicillium marneffei* is an opportunistic human pathogen endemic to Southeast Asia. At 25° *P. marneffei* grows in a filamentous hyphal form and can undergo asexual development (conidiation) to produce spores (conidia), the infectious agent. At 37° *P. marneffei* grows in the pathogenic yeast cell form that replicates by fission. Switching between these growth forms, known as dimorphic switching, is dependent on temperature. To understand the process of dimorphic switching and the physiological capacity of the different cell types, two microarray-based profiling experiments covering approximately 42% of the genome were performed. The first experiment compared cells from the hyphal, yeast, and conidiation phases to identify “phase or cell-state–specific” gene expression. The second experiment examined gene expression during the dimorphic switch from one morphological state to another. The data identified a variety of differentially expressed genes that have been organized into metabolic clusters based on predicted function and expression patterns. In particular, C-14 sterol reductase–encoding gene *ergM* of the ergosterol biosynthesis pathway showed high-level expression throughout yeast morphogenesis compared to hyphal. Deletion of *ergM* resulted in severe growth defects with increased sensitivity to azole-type antifungal agents but not amphotericin B. The data defined gene classes based on spatio-temporal expression such as those expressed early in the dimorphic switch but not in the terminal cell types and those expressed late. Such classifications have been helpful in linking a given gene of interest to its expression pattern throughout the *P. marneffei* dimorphic life cycle and its likely role in pathogenicity.

Human morbidity and mortality linked to fungal infections are increasing. This is a direct result of increasing numbers of immunocompromised patients associated with escalating HIV/AIDS incidence and increasing numbers of immunosuppressed patients because of medical intervention such as cancer treatment and organ transplantation (De Pauw 2001; Dixon *et al.* 1996; McNeil *et al.* 2001; Nielsen and Heitman 2007; Samonis and Bafaloukos 1992). Pathogenic fungi show a polyphyletic distribution and often have as their closest relatives nonpathogenic species, suggesting that the evolution of pathogenicity is likely to show species-specific characteristics (Bowman *et al.* 1992; Bowman *et al.* 1996; Nielsen and Heitman 2007). Despite this, a common theme in fungal pathogenesis is the ability to undergo developmental and morphological transitions during infection in response to the host environment. Dimorphism is one morphological alteration whereby certain fungi can undergo an environmentally induced morphological switch between a hyphal and a yeast growth form. In many dimorphic fungi, one of the strongest inducers is temperature. This switch allows for adaptation to different environmental conditions and has been correlated with the ability to infect, survive, and cause disease in a host (Gow *et al.* 2002; San-Blas *et al.* 2000). Blocking the ability of a fungus to undergo the switch has been shown to limit its virulence (Klein and Tebbets 2007; Lo *et al.* 1997). Examples of pathogenic dimorphic fungi include *Blastomyces dermatitidis*, *Coccidioides immitis*, *Histoplasma capsulatum*, *Paracoccidioides brasiliensis*, *Sporothrix schenkii*, and *Penicillium marneffei*. *P. marneffei* has recently been renamed *Talaromyces marneffei* as part of a kingdom-wide taxonomic reassessment (Samson *et al.* 2011).

*P. marneffei* is an opportunistic pathogen endemic to Southeast Asia and is strongly associated with AIDS (Cooper and Vanittanakom 2008). *P. marneffei* is the only known dimorphic species in the Eurotiales, which includes the large genera of *Aspergillus* and *Penicillium* (Andrianopoulos 2002). *P. marneffei* grows as multinucleate filamentous hyphae at 25° and can undergo asexual reproduction (conidiation) to produce the infectious asexual spores (conidia) (Vanittanakom *et al.* 2006). At 37° *P. marneffei* undergoes a morphological switch to grow as pathogenic uninucleate yeast cells that divide by fission. The dimorphic nature of *P. marneffei* provides an ideal opportunity to tease out key differences between nonpathogenic and pathogenic growth forms, all within the one organism (Andrianopoulos 2002). This process has been significantly aided with the recent completion of the *P. marneffei* genome sequence (Alex Andrianopoulos and William C. Nierman, unpublished data), highlighting gene groups common to dimorphic fungi as well as genes unique to *P. marneffei*.

In the past decade, DNA microarray analysis has proved to be a powerful and cost-efficient means of assaying differential gene expression on a large scale and has provided insight into the mechanisms that regulate processes such as growth, pathogenicity, and antifungal susceptibility (Black and Hare 2000; Garaizar *et al.* 2006; Kadosh and Johnson 2005; Saville *et al.* 2005). In fungi, microarray analysis has been successful in identifying cell-type–specific differential gene expression in dimorphic fungi such as *H. capsulatum* (Hwang *et al.* 2003) and *Coccidioides posadasii* (Johannesson *et al.* 2006). In the dimorphic pathogen *P. brasiliensis*, comparative microarray analysis of mycelial and yeast cells showed that genes upregulated in yeast cells encode proteins implicated in respiratory and metabolic processes as well as a range of transporters (Felipe *et al.* 2005). In contrast, genes involved in cell division and protein catabolism were notably associated with mycelial cells. Interspecies comparisons were also conducted to identify orthologous genes identified in *P. brasiliensis* from the genome sequence of *H. capsulatum* and *C. posadasii* in search of common dimorphic fungi-specific pathways. Common to the pathogenic forms of all three fungi was the expression of orthologous transporters, including calcium, copper, and drug resistance transporters (Monteiro *et al.* 2009).

To gain insight into the physiological capacity of the different growth forms in *P. marneffei*, phase or cell-type–specific gene expression patterns were examined using hyphal and yeast cells, as well as cells of the asexual development apparatus (conidiophore) that generate the infectious conidia. All other studies to date have only examined the hyphal and yeast vegetative growth forms in dimorphic fungi, including those in *P. marneffei* (Lin *et al.* 2012). Further, a second set of microarray experiments was also performed to examine gene expression during the early stages of the switch between the hyphal and yeast growth forms.

These data sets have identified several classes of genes that are likely to be involved in cell-type specification and maintenance, as well as those required for early and late events in cell-type specificity. The differentially expressed genes identified were organized into clusters based on expression patterns and predicted function. Detailed inspection of some of these genes has been helpful in linking temporal expression pattern to function throughout the dimorphic life cycle of *P. marneffei* and, in turn, in identifying the likelihood of a role in pathogenicity. For example, genes that were highly differentially expressed in yeast cells reflected the requirement for adaptation to a stressful environmental, with genes involved in iron acquisition and ergosterol biosynthesis upregulated throughout yeast cell morphogenesis and growth. Expression profiling of all the genes in the ergosterol biosynthetic pathway revealed a unique regulatory pattern for certain steps in the pathway that highlight differences between the cell types. Deletion of the highly differentially regulated *ergM* (encoding C-14 sterol reductase) resulted in severe growth defects, particularly at 37°, and an alteration in antifungal drug susceptibility. Genes upregulated late in yeast cell morphogenesis included those related to adhesion to host tissue, genes involved in the synthesis of core components of the fungal cell wall, and *P. marneffei*–specific genes of unknown function. One gene, *ystA*, was shown to encode a short 68-amino-acid protein that has rapidly diverged under selective pressure and to play a role in germination. A consistent difference between cell types in this study is the differential expression of genes involved in cell wall and membrane biogenesis, highlighting fundamental differences in these structures between the hyphal and yeast cell types.

## Materials and Methods

### Fungal strains and culture conditions

*P. marneffei* strains used in this study are described in Table 1 and were grown on *Aspergillus*-defined medium containing 1% glucose and 20 mM GABA (ANM) (Cove 1966). For microarray experiments, *P. marneffei* was grown at either 25° on ANM plates for asexual development (4 d) or shaken in liquid brain–heart infusion (BHI) (Oxoid) for hyphal growth at 25° (2 d) and for yeast growth at 37° (4 d of growth and then 10 ml was transferred to fresh medium for an additional 2 d). Auxotrophic supplements were added as required. For temperature switching microarray experiments, hyphal cells were transferred to 37° for 6 hr after 2 d of growth at 25°, or yeast cells were moved to 25° for 6 hr after 4 d of growth at 37°.

**Table 1:** *P. marneffei* strains

Name	Strain	Relevant Genotype	Origin
	FRR2161	Wild-type	ATCC 18224 or Borneman *et al.* 2000
G147	SPM4	*niaD1 pyrG1*	Borneman *et al.* 2000
G816	Δ*ligD*::*pyrG^−^*	Δ*ligD niaD1 pyrG1*	Bugeja *et al.* 2012
G809	Δ*ligD*::*pyrG^+^*	Δ*ligD*::*pyrG*^+^ *niaD1 pyrG1*	Bugeja *et al.* 2012
G526	Δ*pkuA pyrG^−^*	Δ*pkuA areA*^Δ^*^DBD^ pyrG1 niaD1*	Bugeja *et al.* 2012
G681	Δ*pkuA*::*pyrG^+^*	Δ*pkuA*::*pyrG*^+^ *areA*^Δ^*^DBD^ niaD1*	Bugeja *et al.* 2012
SBA001	Δ*ystA pyrG*^−^	Δ*ystA pyrG*^-^ *areA*^Δ^*^DBD^ niaD1*	This study
SBA002	Δy*stA pyrG^+^*	Δ*ystA*::*pyrG*^+^ *areA*^Δ^*^DBD^ niaD1*	This study
SBA003	Δ*ystA ystA^+^*	Δ*ystA*::*ystA^+^ pkuA*::*pyrG*^+^ *niaD1*	This study
SBA004	*ystA*^ORF1^-GFP	Δ*ystA pyrG^+^*[pSB7326, *barA^+^)*]	This study
SBA005	*ystA*^ORF2^-GFP	Δ*ystA pyrG^+^*[pSB7338, *barA^+^*]	This study
SBA006	Δ*ystA*, *ystA^M(ORF1)L^*	Δ*ystA pyrG^+^* [pSB7550, *pyrG^+^*]	This study
SBA007	Δ*ystA*, *ystA^M(ORF2)L^*	Δ*ystA pyrG^+^* [pSB7551, *pyrG^+^*]	This study
MPA001	Δ*ergM pyrG^+^*	Δ*ergM*::*pyrG*^+^ Δ*ligD niaD1*	This study
MPA002	Δ*ergM ergM^+^*	Δ*ergM*::*ergM^+^* and *ligD*::*pyrG*^+^ *niaD1*	This study

### Molecular techniques

For genomic DNA isolation, strains were grown in synthetic dextrose (SD) liquid medium supplemented with 10 mM (NH_4_)_2_SO_4_ for 4 d at 25° before mycelia was harvested by filtration through Miracloth, washed with 0.1 M MgSO_4_, dried, and frozen in liquid nitrogen. Genomic DNA was then isolated per standard protocols (Borneman *et al.* 2001). Genotypes were confirmed by Southern blot hybridization after transfer of DNA onto Amersham Hybond N+ membranes using 0.4 M NaOH according to manufacturer’s instructions. Radiolabeled probes (α-^32^P dATP) were added in hybridization solution (50% formamide, 4% SSPE, 1% SDS) at 37°, incubated overnight and washed with 2% SSC at 37° (low stringency) and 0.1% SSC at 65° (high stringency). Radiolabeled probes were made using the random hexamer primer method (Sambrook *et al.* 1989).

RNA for microarray analysis was prepared using the QIAGEN RNAeasy kit according to the manufacturer’s instructions, followed by a LiCl_2_ precipitation. RNA samples for northern blot analysis (10 µg) were separated on 1.2% agarose formaldehyde denaturing gels, transferred onto Amersham Hybond N+ membranes, and analyzed by northern blot hybridization (Sambrook *et al.* 1989).

### Cloning and plasmid construction

The propagation of plasmid DNA was performed by transformation of *Escherichia coli* TOP10 cells (Life Technologies). Cells were grown overnight at 37° on Luria-Bertani broth plates supplemented with 50 μg/ml ampicillin. The *ystA* coding sequence with 5′ and 3′ flanking regions was amplified by PCR (primers AA50 and AA51). The Δ*ystA* construct, in which the coding region was replaced with the *pyrG* selectable marker gene, was generated by fusion PCR (primers AA52, AA53, AA62, AA63, AA64, AA65; pSB7593). The *ergM* coding sequence with 5′ and 3′ flanking regions was amplified by PCR (primers KK1 and KK2). The PCR product was then ligated into the plasmid pALX223 at the *Eco*RV restriction site, resulting in plasmid pMP7459. The Δ*ergM* construct was generated by inverse PCR using primers KK25 and KK26 on plasmid pMP7459. This PCR removed the coding region of the gene. The *pyrG* selectable marker was ligated into the PCR products to generate the deletion constructs (pMP7507). Linearized deletion constructs of *ystA* and *ergM* were transformed into the *P. marneffei* Δ*pkuA pyrG*^-^ and Δ*ligD pyrG* strains, respectively, as previously described (Borneman *et al.* 2001) and selected on uracil free medium.

The *ystA* locus was PCR-amplified from FRR2161 genomic DNA and used to construct two plasmids. A 2.6-kb PCR product incorporating the predicted ORF1 (primers GG73 and EE71) and a 2.8-kb PCR product incorporating ORF2 (primers GG73 and GG72) were separately ligated into pGEM-T Easy. After sequencing, the inserts were excised by a *Cla*I/*Hind*III digest and ligated into pALX190 to generate in-frame fusions with GFP. These fused products were then ligated into pMT1612 RV SK^+^, containing the selectable marker *barA*. These *ystA*-GFP fusion constructs, *ystA*^ORF1^-*GFP* (pSB7326) and *ystA*^ORF2^-*GFP* (pSB7338), were targeted to the 5′ upstream region of Δ*ystA pyrG*^+^, with the presence of *barA* allowing for candidate transformant screening on glufosinate-supplemented medium. Constructs of *ystA* with a mutated ATG in either ORF1 [*ystA*^M(ORF1)L^] or ORF2 [*ystA*^M(ORF2)L^] were generated by inverse PCR (ORF1 invPCR primers GG67 and GG68; ORF2 invPCR primers GG69 and GG70) on *ystA*::*pyrG* targeting vector. Circular mutant constructs were transformed into *P. marneffei* and selected on uracil free medium.

### Library construction

Two independent genomic DNA libraries were constructed to ensure maximal representation of the *P. marneffei* genome. Fungal genomic DNA was partially digested with either *Sau3*AI or *Taq*I. DNA fragments were separated on a 1.5% agarose gel and those ranging from 400 to 600 bp were purified. The size-selected genomic DNA was ligated into pALX223 cut with either *Bam*HI (for the *Sau3*AI library) or *Cla*I (for the *Taq*I library) and chemically competent *E. coli* cells transformed with these libraries. Transformants were selected on ampicillin/X-gal/IPTG plates and screened for white colonies.

### Microarray construction

Independent colonies from both libraries were selected for the construction of the microarrays at the Australian Genome Research Facility (http://www.agrf.org.au/). Colonies (5376) were picked using the BioRobotics BioPick machine and individually placed into 384-well plates containing Luria-Bertani broth. Colonies were then grown overnight and DNA was prepared from a 2-μl aliquot of each culture and used to PCR-amplify the gDNA inserts with M13 forward and reverse primers. The resulting PCR products were purified using Millipore Multiscreen filter plates via a manifold vacuum. The quality and quantity of the purified amplified product were ascertained through agarose gel electrophoresis. Amplified DNA inserts were adjusted to 250 ng/μl in 50% DMSO buffer for printing. The amplified DNA inserts were arrayed in duplicate onto Corning GAPS II aminosilane glass slides using the ESI Virtek Arraying Robot. DNA was spotted using Point Technology split pins. Slides were baked and UV-irradiated to crosslink the DNA.

### cDNA synthesis, labeling, and hybridization

First-strand cDNA synthesis was achieved using the SuperScript Indirect cDNA Labeling System (Invitrogen) according to the manufacturer’s instructions. Total RNA (50 µg) was used in each labeling reaction. Cy3 and Cy5 dyes (Amersham) were coupled to the amino-allyl groups in 0.1 M sodium bicarbonate pH 9.0 buffer for 1 hr in the dark. Unincorporated dyes were removed using QIAquick PCR Purification Kit (Qiagen). Samples were heated at 100° for 2 min in hybridization buffer (25% formamide, 2.5× SSC, 0.01% SDS, 0.32 mg/ml tRNA, and 0.8 mg/ml herring sperm DNA). Samples labeled with Cy3 and Cy5 were then competitively hybridized on microarray slides prehybridized with prehybridization buffer (25% formamide, 5× SSC, 0.1% SDS, and 10 mg/ml BSA). Hybridizations were performed at 42° for 16–20 hr. Slides were washed four times in wash buffer containing decreasing concentrations of SSC and SDS. Dye switch was performed for every pairwise comparison.

### Microarray data analysis

Microarray slides were scanned on either a GenePix 4000B or a 4100A scanner (Axon Instruments), and the resulting images were analyzed with GenePix Pro 4 or 6.1 and Acuity 4.0 to obtain foreground and background intensity estimates for each spot. Statistical analysis was performed using the limma software package (Smyth 2005a). Intensities were background-corrected by the “normexp” method with offset equal to 16 to stabilize the log-ratios of low-intensity probes (Ritchie *et al.* 2007) and then normalized using a global loess curve for each array (Smyth and Speed 2003). The first microarray experiment with eight two-color arrays provided four replicates of conidiating samples and six replicates of hyphal and yeast cells. The second microarray experiment provided four replicates of each temperature switch. Comparisons between the three cell states or responses to temperature switching were extracted from the competitive hybridizations by linear modeling, as previously described (Smyth 2005a). An intercept term was included in the probe-wise linear models to account for probe-specific dye effects (Holloway *et al.* 2006). The duplicate spots for each probe, printed on the top and bottom halves of each slide, were combined using the common correlation method and using an estimated duplicate correlation of 0.50 for the first experiment and 0.72 for the second (Smyth *et al.* 2005b). Differential expression between the three states was assessed using an empirical Bayes moderated analysis of variance (Smyth 2004). The false discovery rate (FDR) was controlled using the Benjamini and Hochberg method. The ternary diagram was produced using the compositions software package (http://www.r-project.org) after first converting the log-fold changes between the states into compositional values representing relative expression. Microarray data are available from the NCBI Gene Expression Omnibus database under accession numbers GSE51109 and GSE51110.

### DNA sequencing and analysis

Sequences of the probes representing genes with differential expression were obtained by sequencing the corresponding plasmid using the M13 forward or reverse primer. A pipeline was created for automated vector sequence trimming and database searches (blastn and blastx) for the sequences of the top 500 differentially expressed clones in the phase-specific microarray and those uniquely differentially expressed in the switching microarray experiment. Blast outputs were then evaluated for hits with an E-value more than 0.001 and manually curated. Clones with ambiguous gene assignment or high E-values were examined further by obtaining the sequence of both flanking DNA regions from the *P. marneffei* genome. Clones showing low-quality sequence or more than one ORF within the insert region were discarded from further clustering analysis (Supporting Information, Table S2 and Table S3).

### Microarray data clustering and gene ontology analysis

Clustering analysis was performed with Multiexperiment Viewer (MeV) 3.1 (Saeed *et al.* 2006) using k-means clustering and Pearson correlation (Table S4 and Table S5). Gene ontology (GO) terms were allocated to each gene represented by the sequenced microarray probes using Blast2GO (http://www.blast2go.com/). These GO assignments were then used to examine significant gene relationships within each cluster using GoTermFinder (http://go.princeton.edu/cgi-bin/GOTermFinder) and statistical significance determined by calculating p-values (p-value < 0.05) using the hypergeometric distribution described by Boyle *et al.* (2004) that takes into account bias in the sample population. The FDR was also calculated (Table S6 and Table S7).

### Gene expression analysis

RNA was extracted using TRIzol Reagent (Invitrogen) and the MP FastPrep-24 bead beater according to the manufacturer’s instructions. RNA was DNase-treated (Promega) before RT-PCR and cDNA synthesis using the DyNaMo cDNA synthesis kit. An increasing number of cycles was used to ensure the amplification was in the exponential phase, and *H3* (histone H3) or *benA* (β-tubulin) were used as a loading control. Microarray analysis results were validated by performing RT-PCR on a subset of differentially expressed genes from independently isolated RNA produced under the same conditions as those for the microarray experiments (PMAA_076130, PMAA_071450, PMAA_065680, PMAA_053710, PMAA_082030, PMAA_082040, PMAA_010220, PMAA_097290, PMAA_0572450, PMAA_040300, PMAA_018640, and PMAA_091310) (Figure S1) using primers listed in Table S1.

### Western blot analysis

Strains were grown at 37° in liquid BHI medium for 6 d. Total protein extraction was performed as described previously (Todd *et al.* 2005) and concentrations were determined using a protein assay reagent (Bio-Rad). Proteins were fractionated using a SDS-PAGE gel and transferred to Hybond ECL. Blots were probed with polyclonal rabbit anti-GFP (1/5000) followed by anti-rabbit IgG HRP (1/4000) (Promega).

### Microscopy

For yeast cells, *P. marneffei* strains were grown on a thin layer of solid heart infusion medium (HI) covering a glass slide resting at an angle in a sterile Petri dish, with one end in liquid HI, and incubated at 37° for 5 d (Borneman *et al.* 2000). Cells were fixed in 4% paraformaldehyde in PME [50 mM piperazine-N,N′-bis(2-ethanesulfonic acid), 1 mM MgSO_4_, 20 mM EGTA pH 6.7] for 30 min at room temperature followed by two washes with PME. Cells were then stained with either fluorescent brightener 28 (calcofluor; final concentration 0.014 mg/ml) or Hoescht 33258 nuclear stain (final concentration 0.14 μg/ml) in Tween 80. The same procedure was followed for hyphal cells, except ANM medium was used and growth occurred for 3 d at 25°. Slides were examined using differential interference contrast and fluorescent optics on a Reichart Jung Polyvar II microscope. Images were captured using a SPOT CCD camera (Diagnostic Instruments) and processed with Adobe Photoshop.

### Germination assay

Conidia (1×10^6^) were added to 500 μl liquid SD [with 10 mM (NH_4_)_2_SO_4_] and allowed to germinate at 25° or 37° for 0, 12, 16, or 24 hr. One hundred cells were counted from each sample and the cell type was recorded as either ungerminated or germinated. Three biological repeats were performed for quantification and error bars represent the SEMs. Data were analyzed using Prism 4.0 (GraphPad Software) and statistical analysis was performed using the R statistics package (http://www.r-project.org/).

### Antifungal drug assay

Minimum inhibitory concentration was determined for the antifungal drugs voriconazole, fluconazole, and amphotericin B for the Δ*ergM* strain and controls using e-test strips (bioMérieux). Agarose medium (4 ml of 0.7%) containing 10^5^ conidia was poured onto a Petri dish containing a 2% agarose solution of the same medium. The e-test strips were then placed on top of the medium and the plates were incubated for 5 d (for control strains) or 10 d (for Δ*ergM* strain) at 25° and 37°. At 25° ANM medium was supplemented with 10 mM (NH_4_)_2_SO_4_, whereas at 37° HI medium was used. Minimum inhibitory concentration was determined from the zone of inhibition according to the manufacturer’s instructions.

## Results and Discussion

### Identification of trends in cell-type specific differential gene expression

At 25° *P. marneffei* grows as multinucleate filamentous hyphal cells. It undergoes asexual reproduction (conidiation) in response to specific environmental cues via a series of cellular differentiation steps culminating in the production of asexual conidia (Boyce and Andrianopoulos 2013). On exposure to 37°, *P. marneffei* cells undergo a morphological switch to uninucleate yeast cells. The hyphal, yeast, and conidial cells are distinct and stable morphological cellular states that represent saprophytic, pathogenic, and infectious cells, respectively, but they also have the capacity to interconvert in response to environmental stimuli. To examine the transcriptional profile of each of these cell states, RNA from three biological replicates was isolated and used in pairwise combinations to probe a custom-generated microarray. This microarray was produced by spotting inserts from two size-selected, random fragment genomic libraries containing short (400-600 bp) inserts from the type strain of *P. marneffei* (FRR2161) (see *Materials and Methods* section). The rationale for this approach was that given there was no *P. marneffei* genome sequence available at the time, the genome size is small (estimated to be approximately 26–30 Mb) (Yuen *et al.* 2003) and gene-rich [fungal genomes from related species showed that intergenic regions are relatively short, genes do not have many introns, and those introns that exist are generally short (50–200 bp)] (Galagan *et al.* 2005; Kupfer *et al.* 1997), then most short random fragments would cover transcribed regions and rarely overlap two independent transcriptional units. This was preferred over a cDNA-based approach to probe design because it was subject to less bias. Such an approach has been used previously for other microorganisms (DeRisi *et al.* 1997; Hwang *et al.* 2003; Monteiro *et al.* 2009).

**Figure 1:**
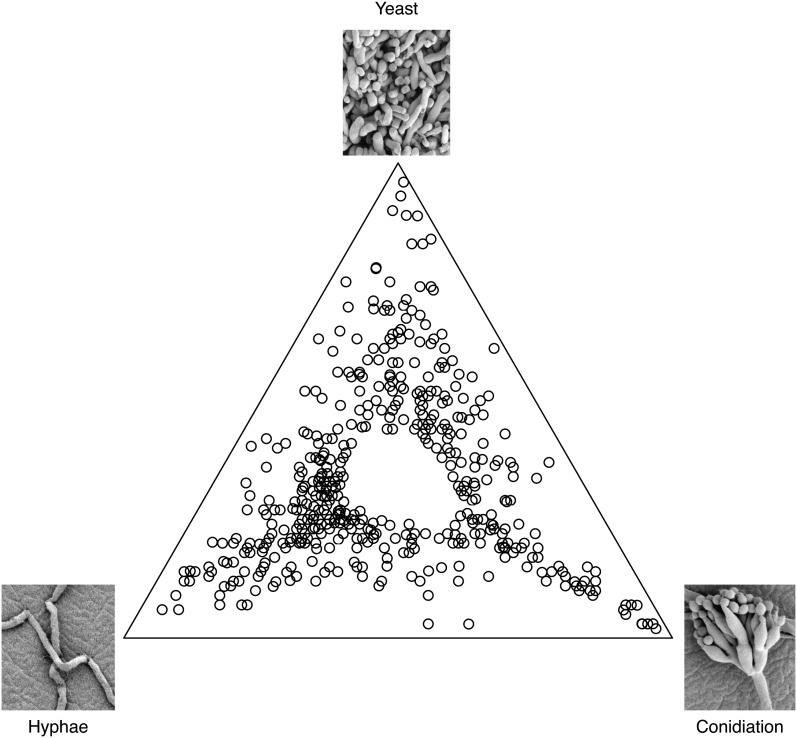
Cell-state–specific differential gene expression in *P. marneffei*. Microarray analysis was conducted with wild-type *P. marneffei* under three growth conditions, 25° on ANM plates for asexual development (4 d), agitated in liquid BHI for hyphal growth at 25° (2 d), and yeast growth at 37° (4 d of growth followed by 10 ml transferred to fresh medium for an additional 2 d). The data set represents the top 500 differentially expressed genes. Points closest to the top corner represent genes with maximum log expression in the yeast phase. Points in the bottom right corner are genes most highly expressed during conidiation, and points at the bottom left corner of the triangle represent genes differentially expressed highest in the hyphal phase. Points between two corners, along the sides of the triangle, depict clones upregulated in two conditions with respect to the one in the opposite corner. Probes expressed evenly among the three states were omitted from the data set used to generate this representation, accounting for the absence of spots in the center of the triangle.

One of the goals of this study was to identify a set of genes that represent cell-type–specific markers to be used for genetic screens and studies of cell function. A second goal was to identify potentially interesting genes for further characterization. Random probes from the libraries (5376) along with 38 probes for 34 previously characterized genes, spotted in duplicate, were arrayed and used in these experiments. More than 38% of the microarray probes were found to show statistically significant differences in expression levels between the three cell states (FDR < 0.05). The 500 most significant clones were selected for further study. Of the top 500 probes, 31.2% were found to be hyphal-specific, 18.2% were conidiation-specific, and 20.2% were yeast-specific (Figure 1). Here, we define hyphal-specific probes to be those with at least 50% higher expression in hyphal cells compared to either of the other two states (similarly for conidiation-specific and yeast-specific probes). The data successfully show dynamic changes in gene expression profiles among the three states and clearly identify probes expressed predominantly in one cell state. Such probes correspond to genes with possible roles in cell-type maintenance, whether they are morphological or physiological.

Clones representing the top 500 differential expressed probes were sequenced and used in BLAST searches (blastn and tblastn) against the available fungal sequence databases in Genbank (http://blast.ncbi.nlm.nih.gov/Blast.cgi). Subsequently, blastn searches against the *P. marneffei* FRR2161 genome sequence were performed when it became available. Microarray clones were omitted from the data set if BLAST searches resulted in no significant *P. marneffei* hits using an E-value cut-off <1×10^−6^. These clones represent either missing sequences from the *P. marneffei* genome project or contaminating clones in the library. In cases in which multiple clones identified the same gene, duplicates were removed. This resulted in a set of 400 unique differentially expressed *P. marneffei* genes under the three tested conditions. Based on this frequency of “false” or duplicate clones, the entire microarray is predicted to represent 4301 unique *P. marneffei* genes, which is 42% of the predicted genes in the genome. Microarray analysis results were validated by RT-PCR on a subset of differentially expressed genes (Figure S1). The trends in expression between the microarray data and the RT-PCR were highly correlative for the genes identified as yeast-enriched and conidiation-enriched, but less so for the genes identified as hyphal-enriched (Figure S1).

**Figure 2:**
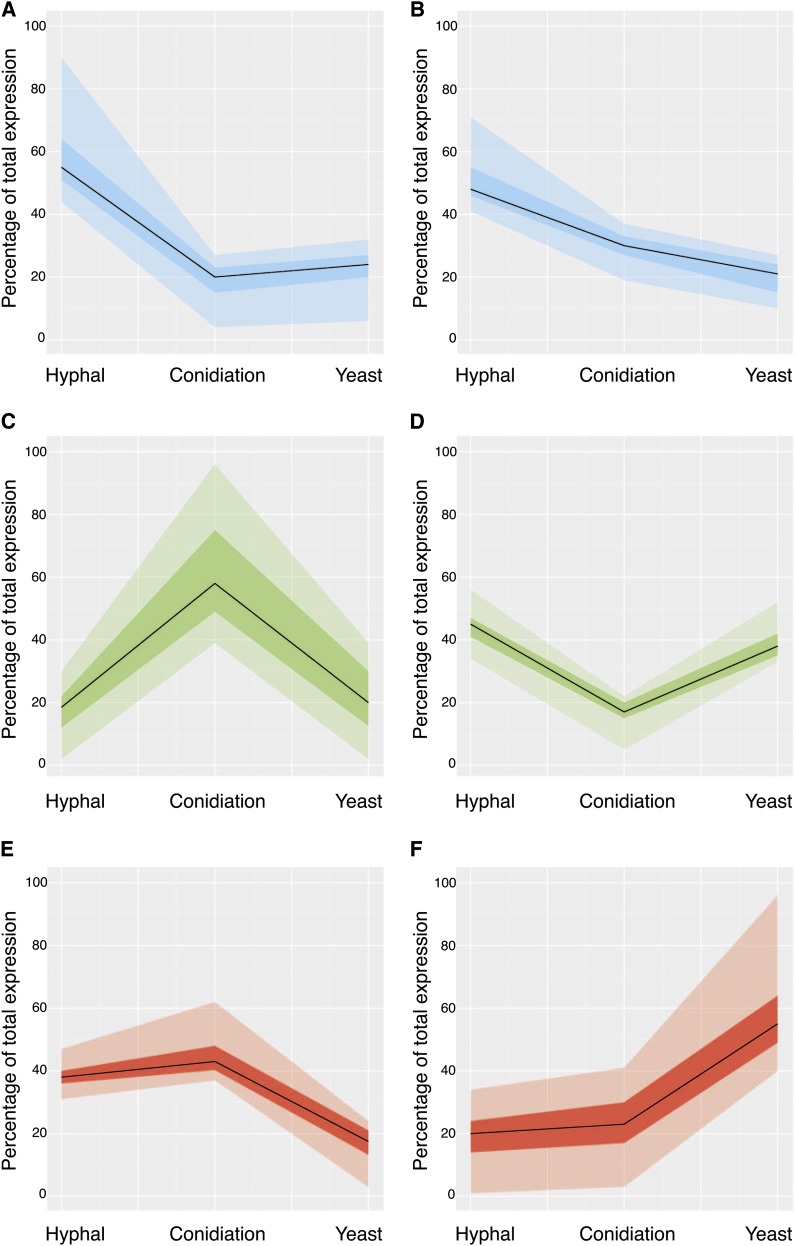
Gene expression clustering pattern in *P. marneffei* cell states. Clustering analysis was performed using the top 400 unique genes differentially expressed in hyphal, yeast, or conidiation conditions with MeV Six clusters were created using k-means clustering with Pearson correlation. The median of gene expression under each condition is represented as a continuous black line. The dark shaded region in each graph represents the differential expression values that lie within the first and third quartiles of the cluster. The lighter shading extends the spread of data from zero to the fourth quartile. (A) Genes upregulated in hyphal cells. (B) Genes upregulated in hyphal cells, followed by conidiation, and genes downregulated in yeast cells. (C) Genes upregulated during conidiation. (D) Genes upregulated in both hyphal and yeast cells with respect to conidiation. (E) Genes upregulated in hyphae and during conidiation, with respect to yeast cells. (F) Genes upregulated in yeast cells.

Trends in gene expression were examined by k-means clustering using MeV (Saeed *et al.* 2006). The data were clustered into six general expression profiles using Pearson correlation (Figure 2). Gene clusters reflect genes that are exclusively expressed or upregulated in one state, as well as those coregulated in two states (represented as percentages of total expression). Because both hyphal growth and conidiation occur at 25°, and because conidiation begins only after a period of hyphal growth, the presence of genes commonly upregulated in both states is expected. Genes that showed increased expression in hyphal and yeast cells relative to conidiation are likely to represent general cellular processes required for vegetative growth as opposed to those specific for the differentiation processes involved in conidiation. Interestingly, there is an underrepresentation of genes downregulated in hyphal cells and upregulated during both conidiation and in yeast cells. This may be indicative of the difference in specificity involved in reproduction by fission in yeast cells and differentiation during conidiation. Binary fission involves the segregation of replicated genetic material to the poles of a cell that then splits into two identical cells. However, asexual reproduction during conidiation is performed in a budding fashion with the specialized differentiation of uninucleate cells (Borneman *et al.* 2000; Clutterbuck 1969). Genes that have opposing expression in these cell states may provide information regarding what gene groups govern these differences in cell division. As a way of addressing what categories of genes are within each cluster and whether any of these are enriched for a particular state, GO terms were assigned to each gene represented by the microarray probes, and these were curated further by a qualitative literature-based approach. These GO assignments were then used to examine significant gene relationships within each cluster (p-value < 0.05) (http://go.princeton.edu/) (Figure S2), and they highlighted, in particular, the enrichment of gene groups during conidiation (Table 2).

**Table 2:** Gene groups upregulated during conidiation

GO ID (cluster uncorrected p-value) and description	Gene ID	Percentage of Total Expression			BLASTX Description and Role	Reference
		Hyphal	Conidiation	Yeast		
GO:0070317 (0.003): negative regulation of G0-to-G1 transition	PMAA_096670 (*smD3* ortholog*)*	16	56	29	Small nuclear ribonucleoprotein: part of the heteroheptameric complex that is essential for spliceosomal U1, U2, U4, and U5 snRNPs. Involved in nuclear mRNA splicing	Roy *et al.* (1995); Bordonne (2000)
	PMAA_042130 (*nimE*)	28	52	20	G2/M-specific cyclin: regulates activity of cyclin-dependent protein kinase processes	O’Connell *et al.* (1992)
	PMAA_017210 (*erg13* ortholog)	20	43	37	Hydroxymethylglutaryl-CoA synthase: catalyzes the second step of mevalonate biosynthesis	Parks and Casey (1995)
GO:0008643 (0.008): carbohydrate transport	PMAA_049040	17	66	17	MFS monosaccharide transporter	
	PMAA_047000	12	78	10	Sugar transporter	
	PMAA_049550	21	60	19	Sugar transporter	
	PMAA_059270	29	50	22	MFS alpha-glucoside transporter	
GO:0019954 (0.035): asexual reproduction	PMAA_081680 (*wetA)*	24	54	22	Developmental regulatory protein: activates conidiation-specific gene expression	Boylan *et al.* (1987); Marshall and Timberlake (1991);
	PMAA_082040 (*arpA)*	3	94	3	Scytalone dehydratase: involved in conidial pigment biosynthesis	Tsai *et al.* (1999)
	PMAA_075300 (*brlA)*	3	91	7	C_2_H_2_ type conidiation transcription factor: regulation of conidiophore development	Adams *et al.* (1988)
	PMAA_006690 (*dfgE*)	21	56	23	Cell wall glycosyl hydrolase: required for cell wall synthesis in bud formation and filamentous growth	Kitagaki *et al.* (2002)

### Transcription profiles of nonpathogenic hyphal and pathogenic yeast cells during early and late cell morphogenesis

**Figure 3:**
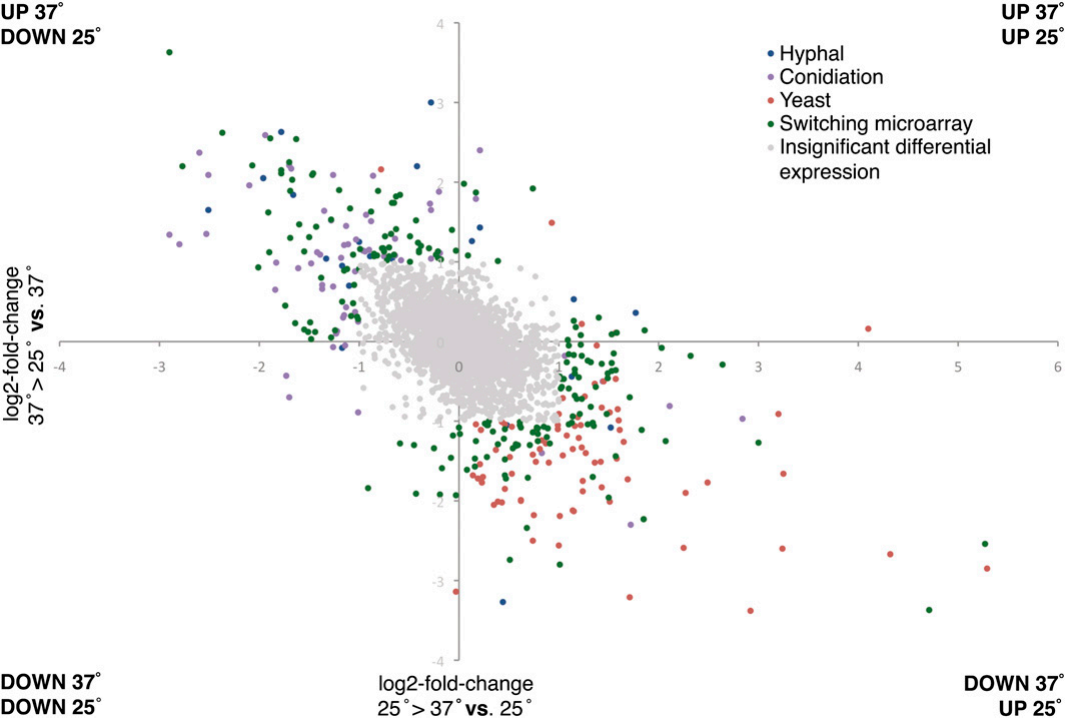
Differential gene expressions during a temperature-induced morphological switch. Temperature switching microarray experiments involved the transfer of hyphal cells to 37° for 6 hr after 2 d of growth at 25° and the transfer of yeast cells to 25° for 6 hr after 4 d of growth at 37°. Log2 fold change during 25° to 37° *vs.* 25° represents the expression of genes at 25° compared to those transferred to 37° for 6 hr and Log2 fold change during 37° to 25° *vs.* 37° represents the expression of genes at 37° compared to those transferred to 25° for 6 hr. Color-coding is based on comparison to three-phase–specific microarray (Figure 1). Blue probes identified the highest expression during late hyphal cell growth compared to other cell states and red probes identified the highest expression during late yeast cell growth. Green clones represent probes that showed significantly different expression between hyphal and yeast cells during the first 6 hr and not during later development. Probes shaded gray were omitted from subsequent analysis and showed no significant difference between the cell states in expression during the first 6 hr of growth.

Microarray analyses of hyphal, yeast, and conidiation cell states reflect gene expression differences late in morphogenesis and development and are not necessarily representative of the transcriptional events during the switch from one temperature to another and the initial events of cell-type establishment. To examine differential gene expression early in the transition between hyphal and yeast cells, microarray analysis was performed using RNA from cells grown at 25° or 37° and then either maintained at this temperature or switched to the alternate temperature for an additional 6 hr (see *Materials and Methods* section). The resulting data were converted to log2 ratios of expression after temperature switching *vs.* maintenance at a constant temperature (Figure 3). The initial data set showed substantial changes in gene expression across many probes (2.4% upregulated and 2.1% downregulated by two-fold or more during 25° to 37° switch, 2.5% upregulated and 2.7% downregulated by two-fold or more during 37° to 25° switch). From this larger data set, clones that showed less than a two-fold increase or decrease in expression were omitted from further analysis, as were known duplicates, and the remaining values were then compared to expression later during that state. After which, clones representing differentially expressed probes unique to the switching microarray (73 genes) were sequenced and identified in the same manner described in the three-state microarray. From this new subset of 473 genes, 31.6% of the probes were differentially regulated in both the three-state microarray and the switching microarray experiments. Results indicate that 43% of the genes are upregulated and 57% are downregulated by two-fold or more during the 25° to 37° switch, and 42% are upregulated and 58% are downregulated by two-fold or more during the 37° to 25° switch.

**Figure 4:**
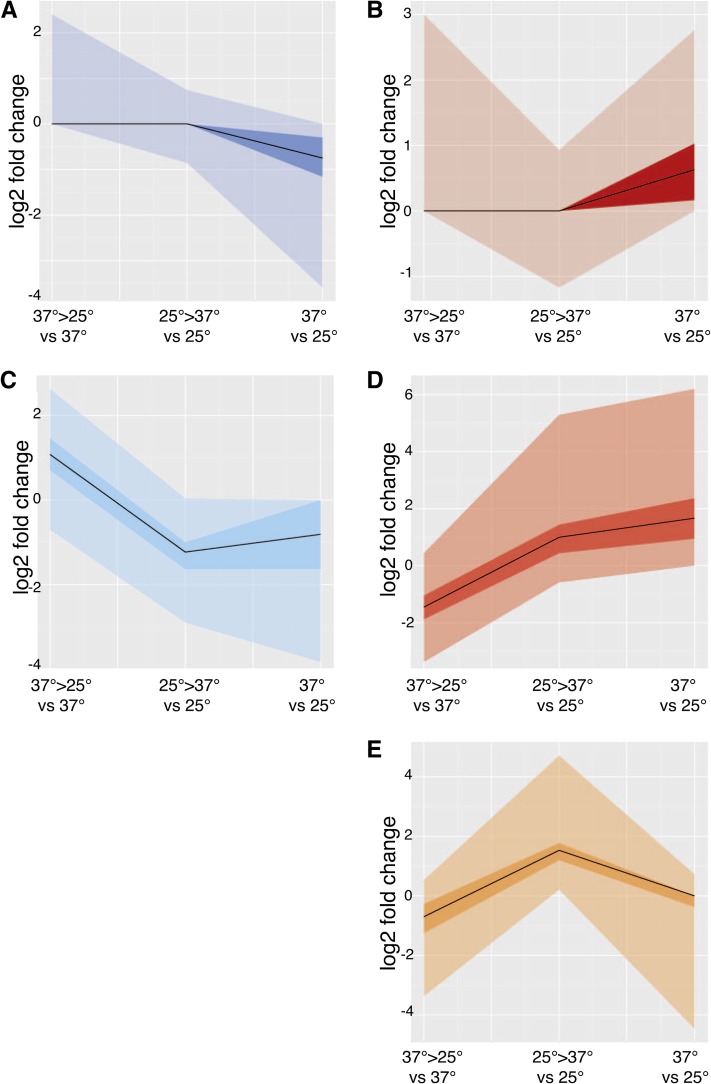
Early and late hyphal and yeast gene expression clustering. Clustering analysis was performed on genes expressed during early and/or late cell-state specific growth with MeV using k-means clustering. Six clusters were created using Pearson correlation, two of which were manually merged because they displayed similar trends. The median of gene expression under each condition is represented as a continuous black line. The dark shaded region in each graph represents the differential expression values that lie within the first and third quartiles of the cluster. The lighter shading extends the spread of data from zero to fourth quartile. X-axis condition 37° to 25° *vs.* 37° shows cells that were transferred to 25° from 37° for 6 hr and compared to 37°. Condition 25° to 37° *vs.* 37° shows cells that were transferred to 37° for 6 hr and compared to 25° gene expression. Finally, condition 37° *vs.* 25° shows cells grown at 37° for 4 d (yeast cells) compared to cells grown at 25° for 2 d (hyphal cells). (A) Genes upregulated in late hyphal cells. (B) Genes upregulated in late yeast cells. (C) Genes upregulated in the switch to 25° and that stayed upregulated in late hyphal cells or remained evenly expressed in late hyphal and yeast cells. (D) Genes upregulated during the switch to 37° and that remained upregulated in late yeast cells. (E) Genes that are upregulated during the switch to 37° and upregulated in late hyphal cells.

To examine the overlap between the expression patterns for the cell-state–specific genes and those identified during the switch from 25° to 37° and vice versa, k-means Pearson correlation testing was performed in MeV using log2-fold change values for all data sets. This analysis allowed for the identification of early and late expressed genes in hyphal and yeast cells. The data were clustered into six expression profiles, with two similar clusters showing genes with increased expression in early and late hyphal cells being manually combined (Figure 4), and GO terms were allocated in a manner previously described (see *Materials and Methods* section). Genes upregulated solely during the switch from 37° to 25° clustered with those that were differentially upregulated late in hyphal growth compared to late yeast cell expression, together accounting for 20% of all the genes (Figure 4C). The clustering showed that 36% of the genes were highly expressed only late in hyphal growth (Figure 4A) and 4% were upregulated only during the switch from 25° to 37° (Figure 4E). An additional cluster of 16% of the genes was upregulated during the switch to 37° and remained upregulated late in yeast cell growth (Figure 4D). Finally, the 24% of genes upregulated only late in yeast cell growth grouped together (Figure 4B). A consequence of clustering the data set into six expression groups, as opposed to binning probes in all possible patterns of gene expression, is that some genes designated as outliers, in the zero to fourth quartiles, may in fact represent genes with underrepresented rare patterns of gene expression (Figure 4).

### Genes upregulated during early and late hyphal cell growth are involved in apical growth and cell wall integrity

Despite being clustered with genes upregulated primarily in established hyphal cells, the homolog of the fungal-specific Ras small monomeric GTPase, *rasB*, is upregulated during the switch to hyphal cells and throughout hyphal development. Ras GTPases have characterized roles in cell polarization and morphology in numerous pathogenic and nonpathogenic fungi as well as other eukaryotes (Boyce *et al.* 2005; Lengeler *et al.* 2000). Deletion of the *rasB* ortholog in *Aspergillus fumigatus*, a prominent monomorphic opportunistic fungus that grows filamentously, results in decreased germination and growth rates, increased hyphal branching, and a loss of virulence (Fortwendel *et al.* 2005). In *Neurospora crassa*, the *rasB* homolog, *NC-ras2*, regulates cell wall synthesis, apical growth of hyphal cells, and conidial formation (Kana-uchi *et al.* 1997).

Another gene highly expressed during early and late hyphal cell growth encodes an ortholog of the GPI-anchored protein Ecm33, which is involved in apical growth and cell wall integrity in *A. fumigatus* and *S. cerevisiae*, among others (Latgé 2007; Pardo *et al.* 2004). *S. cerevisiae* Ecm33 is important for cell wall organization and deletion results in a weakened cell wall and associated glycosylation defects. Ecm33 mutants have defects in the construction of the cell wall mannoprotein outer layer and secrete increased levels of 1,6-β-glucan linked proteins into the growth medium (Pardo *et al.* 2004). Another gene in this group is an ortholog of the *S. cerevisiae BUD4* gene. The GTP binding protein Bud4 is important for apical bud growth and is essential for axial budding pattern and septin organization. A Bud4 ortholog also has morphogenetic roles in *Aspergillus nidulans*, where it is involved in septum formation in hyphae and conidiophores (Si *et al.* 2012).

### Genes upregulated during conidiation play integral roles in asexual development

Conidiation in many filamentous fungi begins with the establishment of a specialized foot cell from which a differentiated aerial stalk extends. Conidia are then produced either directly or after the differentiation of one or more layers of sterigmata (metula and phialide) cells (Cole 1986). In *P. marneffei*, a metula buds off the aerial stalk, phialides bud from the metula, and then a chain of uninucleate conidia sequentially bud off each phialide (Borneman *et al.* 2000; Boyce and Andrianopoulos 2013). Genes identified as upregulated in the conidiation samples are consistent with what is known about the molecular control of conidiation in *P. marneffei* and reinforce the similarities with monomorphic filamentous fungi, such as *A. nidulans*. GO term clustering of these genes highlighted gene groups previously characterized with roles in asexual reproduction (Table 2). For example, orthologuous genes (*smdC*, *nimE*, and *erg13*) involved in the negative regulation of the G0-to-G1 transition, which coincides with conidial dormancy and cell-cycle arrest in the G1 phase (Roy *et al.* 1995; Bordonne 2000; O’Connell *et al.* 1992; Parks and Casey 1995), were upregulated. Important to the long-term survival and dormancy of asexual propagules in filamentous fungi is their pigmentation via dihydroxynaphthalene (DHN) melanin biosynthesis. The conidial pigment biosynthesis gene *arpA*, encoding scytalone dehydratase, showed the second highest conidiation-specific gene expression relative to hyphal and yeast cells and clustered with other “asexual reproduction” genes in the GO term clustering (p-value = 0.035) (Table 2). In *A. fumigatus*, *arp1* is a member of a developmentally regulated pigment biosynthesis gene cluster and shows sequence similarity to scytalone dehydratase of the DHN-melanin cluster identified in other fungi (Tsai *et al.* 1998), and *arp1* mutants produce conidia with a pigmentation that is red-pink rather than blue-green (Tsai *et al.* 1997). Similar to *A. fumigatus*, the *P. marneffei* ortholog of *arpA* is located in a putative DHN-melanin pigment biosynthesis cluster. The biosynthesis of melanin has been associated with increased conidial survival and plays a role in pathogenicity, aiding the fungus in survival and in escaping host defenses (Kwon-Chung *et al.* 1982; Wang et al. 1995; Wheeler and Bell 1988).

In *A. nidulans*, *A. fumigatus*, and *Aspergillus oryzae*, the *wetA* and *brlA* genes are required for conidiation (Mah and Yu 2006; Ogawa *et al.* 2010; Yu 2010). Together with *abaA*, deletion of these genes blocks conidiation at different stages of development but leaves hyphal growth unaffected. Induction of *brlA* alone is sufficient for rudimentary conidiation to occur and *brlA* mutants are able to form stalks, but these fail to undergo any further differentiation (Adams *et al.* 1988). The *abaA* mutants produce aberrant phialides that fail to produce conidia, whereas *wetA* mutant strains produce conidia, but these fail to mature and eventually autolyze (Adams *et al.* 1988; Clutterbuck 1969; Ogawa *et al.* 2010; Timberlake *et al.* 1987). Similarly, the *P. marneffei brlA*, *abaA*, and *wetA* orthologs control various stages of differentiation during asexual development, supporting the conserved roles of these key central regulatory pathway genes (Borneman *et al.* 2000; Anthony R. Borneman and Alex Andrianopoulos, unpublished data).

### Genes upregulated during late stages of hyphal growth are involved in reproduction, hyphal morphogenesis, and cell polarity

**Figure 5:**
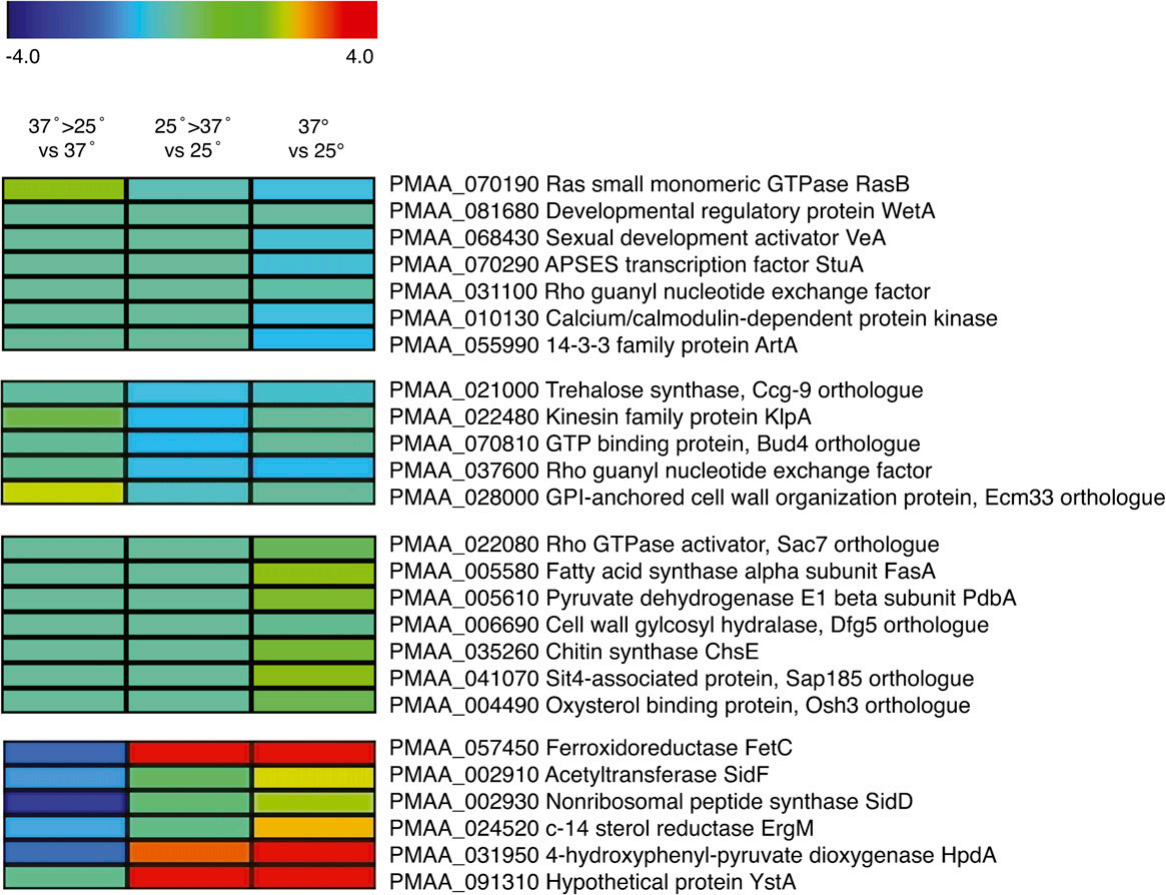
Cell-state–specific expressed genes of interest. Differential expression heat maps of representative genes selected from each cluster, most of which have characterized roles in other fungi. Expression of genes from *P. marneffei* grown at 37° then transferred to 25° for 6 hr compared to 37° (37°>25° vs 37°) or grown at 25° then transferred to 37° for 6 hr compared to 25° (25°>37° vs 25°). In addition, gene expression from cells grown at 37° for 6 d (yeast cells) compared to cells grown at 25° for 2 d (hyphal cells) is shown.

At 25°, hyphal growth of *P. marneffei* begins with the germination of dormant conidia. During germination isotropic growth is activated, followed by the establishment of a polarized axis and germ tube extension. Apical growth of the germ tube culminates in the formation of radiating septate hyphae that branch subapically (Andrianopoulos 2002). Comparing expression profiles of genes differentially expressed in the phase-specific microarray and the switching microarray revealed a cluster of genes that were upregulated in late hyphal growth relative to all other growth conditions (Figure 4). GO analysis grouped a subset of these genes as being involved in developmental processes of reproduction and the generation of precursor metabolites and energy (Figure S2). Further analysis highlighted additional genes fundamental to hyphal morphogenesis and cell polarity (Figure 5).

As alluded to earlier, it is not unexpected that genes upregulated in hyphal cells relative to yeast cells have roles in asexual (conidiation) and sexual reproduction. The gene encoding the APSES transcriptional regulator of mating and asexual reproduction StuA was upregulated late during hyphal cell growth. In *A. nidulans*, *stuA* acts as a modifier of the central regulatory pathway affecting conidiophore patterning and differentiation and is required for sexual reproduction (Clutterbuck 1969). In *P. marneffei*, *stuA* has been shown to be required for the correct spatial organization of conidiophores, with deletion of *stuA* resulting in spore production despite a lack of distinct metula and phialide cell differentiation (Borneman *et al.* 2002).

Appropriate polarity establishment is fundamental to normal cell differentiation and, in turn, hyphal morphology. Present in this cluster is the 14-3-3 family protein encoding gene *artA* (Figure 5). In *A. nidulans*, ArtA plays a role in hyphal morphogenesis, with overexpression causing delays in conidial germination and germ tube establishment (Kraus *et al.* 2002). Also present in this cluster are genes encoding a kinesin family protein and dynactin. Polarized growth requires the delivery of secretory vesicles via microtubules to the hyphal apex and back to the cell center, and vesicle delivery acts as a rate-limiting factor for hyphal tip extension. The microtubule motors kinesin and dynein cooperatively deliver secretory vesicles via microtubules and are likely to be important for maintaining continuous growth (Steinberg 2011). Additionally, the hyphae-specific upregulation of a calcium/calmodulin-dependent protein kinase–encoding gene is consistent with the requirements of cell polarity and apical growth. Large quantities of calcium are essential at the apex of growing hyphal cells, and the calmodulin-calcineurin-Crz1 signaling pathway has been shown to upregulate an array of target genes as a response to increased cytosolic calcium levels (Yoshimoto *et al.* 2002). In *A. fumigatus*, the calcium/calmodulin-dependent kinases and calcineurin catalytic subunit *cnaA* mutant showed dramatic hyphal defects, including decreased apical extension and polarized growth (Steinbach *et al.* 2006).

### Genes upregulated early during yeast cell morphogenesis and throughout yeast growth include those involved in nutrient assimilation and membrane fluidity

The cluster of greatest interest with respect to pathogenicity defines genes that, compared to other cell stages, are highly expressed during the switch to 37° and persist into the yeast cell stage. This cluster, including a series of iron acquisition and assimilation genes, is expressed in the initiation and establishment of yeast cells and, in turn, is likely to have roles in the pathogenic potential of *P. marneffei* (Figure 5).

Iron is essential for the survival of all eukaryotes and most prokaryotes. On microbial infection, the host withholds free iron through sequestration, reducing available free iron within the blood and extracellular fluids of the host (Philpott 2006; Weinberg 2009). Thus, the challenges for invading pathogens become finding ways to obtain and assimilate iron from its host (Dancis *et al.* 1990). In iron-limiting conditions, such as within macrophages, fungi use high-affinity uptake systems, including reductive iron assimilation and siderophore-mediated iron uptake system (Johnson 2008; Kosman 2003). Multicopper ferrioxidase, FetC acting in conjunction with permease FtrA in the reductive iron assimilation pathway to take-up ferrous iron, shows higher gene expression throughout yeast cell initiation and growth compared to hyphal cells (Figure 5). This was also the case with siderophore biosynthetic genes *sidF* and *sidD*. In conditions of low iron, many fungi secrete siderophores, low-molecular-mass ferric iron–specific chelators, to mobilize and transport iron (Schrettl *et al.* 2004; Timmerman and Woods 1999), resulting in siderophore function being considered an important virulence factor in other pathogenic fungi (Haas 2003) and a potential iron-specific target for antifungal drugs (Miethke and Marahiel 2007; Roosenberg *et al.* 2000).

**Figure 6:**
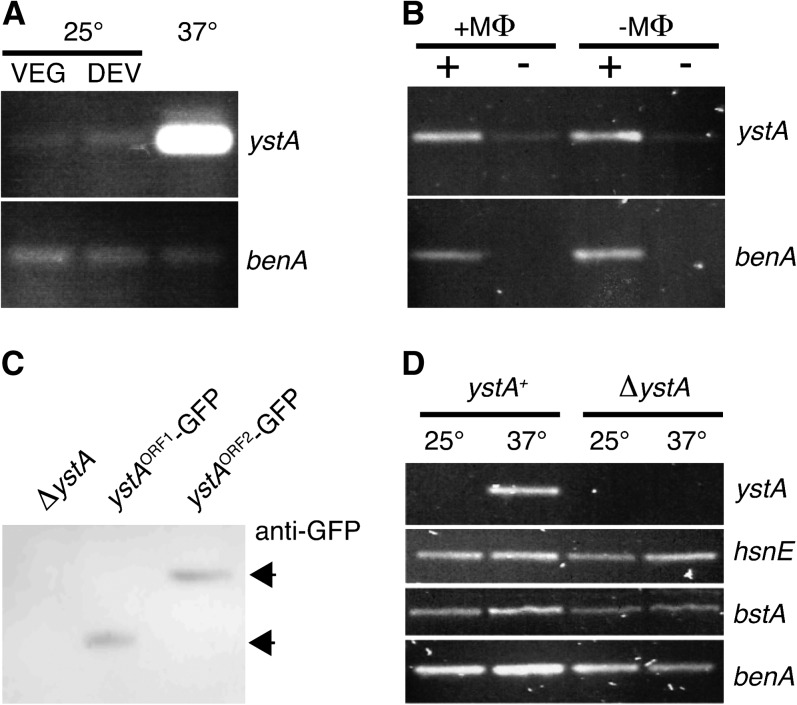
Expression from the 1E11 region in *P. marneffei*. (A) RT-PCR using *P. marneffei* RNA extracted from vegetative hyphal cells growing at 25° for 2 d (VEG), cells undergoing asexual development at 25° after 7 d (DEV), and yeast cells growing at 37° for 2 d using *ystA*-specific primers. Primers for the β-tubulin–encoding *benA* gene were used as a control. (B) RT-PCR using *P. marneffei* RNA extracted from yeast cells growing in J774 mouse macrophages (+MΦ) or in macrophage cell culture medium (−MΦ) for 24 hr using *ystA*-specific primers. To assess the amount of contaminating genomic DNA in the samples, both reverse-transcriptase (+) and no reverse-transcriptase (−) reactions were performed; *benA* was used as a control. (C) Western blot analysis using total cell extracts from *P. marneffei* yeast cells of the *ystA* deletion strain (Δ*ystA*) and strains containing either the *ystA* ORF1 or the ORF2 GFP fusions. Cells were grown at 37° for 2 d and then extracts were fractionated on an SDS-PAGE gel, blotted, and Western blotted with a polyclonal anti-GFP primary antibody and an anti-rabbit HRP secondary antibody. Polyclonal anti-β-tubulin was used as a control. (D) RT-PCR expression analysis of *ystA* and the flanking *hsnE* and *bstA* genes in the wild-type and *ystA* deletion strain using RNA extracted from vegetative hyphal cells growing at 25° for 2 d or yeast cells growing at 37° for 2 d.

Although most of the probes on the microarray identified genomic sequences with readily identifiable gene annotations, several were either intergenic or had low coding potential, making gene annotation difficult. Such probes might represent genes that encode novel short proteins or long noncoding RNAs. One of the most differentially expressed probes was 1E11, which showed very high levels of expression throughout yeast cell growth but no expression during the hyphal phase (Figure 6A). The 1E11 probe is between a gene encoding a second histone H4 (*hsnE*) and a gene encoding a GPI maturation protein (*bstA*) (Figure S2). Northern blot analysis using RNA isolated from hyphal cells grown at 25° and yeast cells at 37° identified ∼850 nt RNA that is transcribed from the opposite strand to *hsnE* (Figure S3). A combination of 3′ RACE and RT-PCR identified the boundaries of this transcript, which contained three short ORFs (encoding 68, 66, and 69 aa) and potentially a long 3′ UTR if any of these ORFs encode a protein (Figure S3). RT-PCR using RNA derived from *P. marneffei* isolated from macrophages showed that this transcript was as abundant under these conditions as in yeast grown *in vitro*. As such, the gene was named *ystA* (yeast-specific transcript A) (Figure 6B).

Sequence comparisons identified *ystA* homologous sequences in the closely related monomorphic fungus *T. stipitatus*, but not in other Eurotiales fungi such as *Penicillium chrysogenum* or *A. fumigatus*. Using the flanking genes, this region was cloned from another closely related fungus, *Penicillium funiculosum*, and sequencing showed that *ystA* was also conserved. Consistent with this, genes across this region are syntenic in the closely related species and disrupted in *P. chrysogenum* and *A. fumigatus* (Figure S2). Alignment of the *ystA* region showed that only ORF2 was conserved. To assess if any of these ORFs are translated, gene fusions with the GFP-encoding gene were generated and integrated into the *P. marneffei* genome either at the *ystA* locus or at the *pyrG* ectopic locus (*Materials and Methods* section). Western blot analysis using a monoclonal anti-GFP antibody identified a single fusion protein of the correct estimated size for each ORF1 and ORF2 in protein extracts from *P. marneffei* grown at 37°, suggesting that both the *trans*-species–conserved ORF2 and ORF1 encode proteins (Figure 6C).

**Figure 7:**
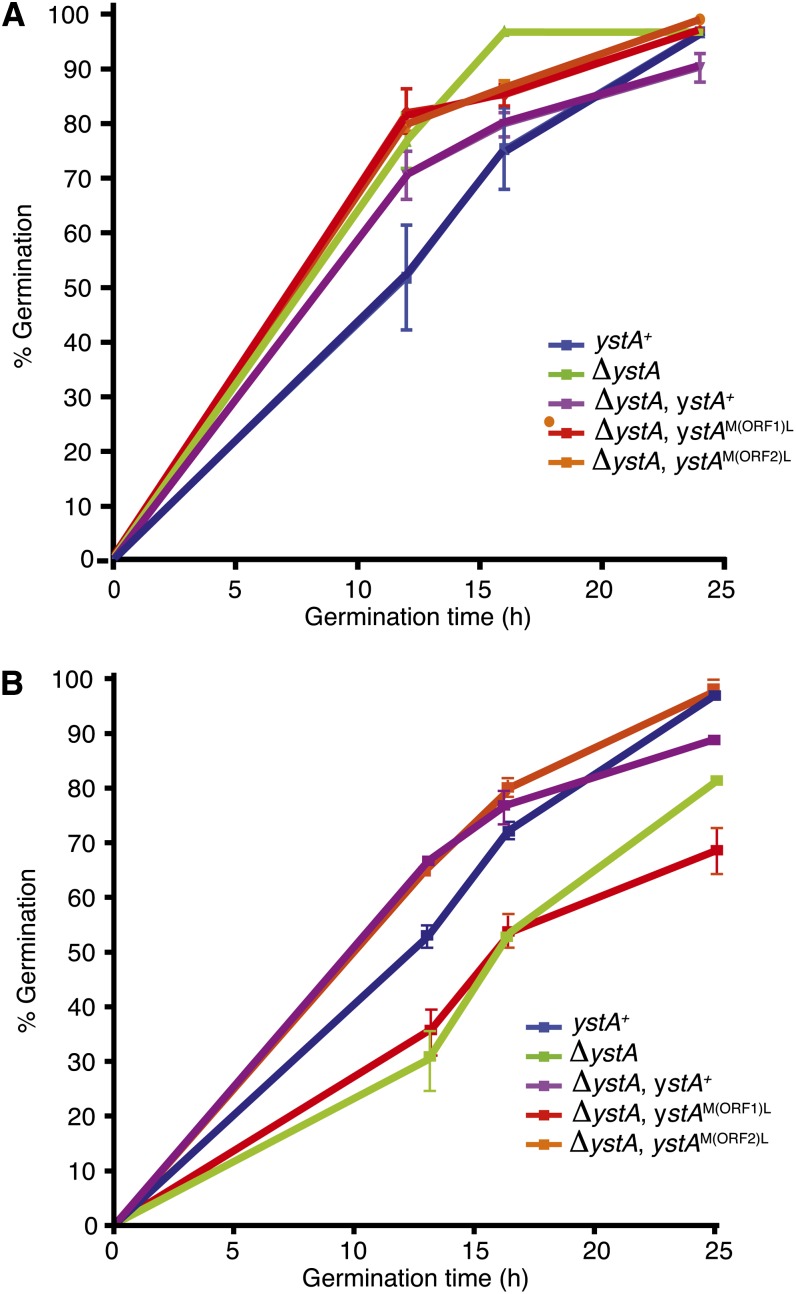
Deletion of *ystA* delays germination at 37°. Time course measuring conidial germination of the wild-type (*ystA*^+^), *ystA* deletion (Δ*ystA*), and complemented *ystA* deletion (Δ*ystA*, *ystA*^+^) strains, as well as two strains carrying point mutations of the predicted initiator ATG of either ORF1 [Δ*ystA*, *ystA*^M(ORF1)L^] or ORF2 [Δ*ystA*, *ystA*^M(ORF1)L^] at 25° (A) and 37° (B). Conidia (1×10^6^) from each strain were inoculated into SD medium at 25° or BHI at 37° in a 24-well microtiter plate and incubated at the appropriate temperature for 0, 12, 16, or 24 hr. One hundred cells at each time point were microscopically examined to assess their germination status. The experiment was performed in triplicate and the error bars represent SEM.

To understand the function of *ystA*, a deletion construct was generated that removes the entire transcribed region and transformation of the G526 strain yielded a number of transformants in which the locus had been deleted (as verified by Southern blot hybridization; data not shown). Phenotypic analysis showed that Δ*ystA* strains were not substantially different from the *ystA*^+^ controls for germination at 25° (Figure 7A) or for hyphal growth and asexual development (data not shown). In contrast, Δ*ystA* strains showed a delay in germination at 37° *in vitro* (Figure 7B). The *ystA* locus was deleted in a second strain of *P. marneffei* (G147) and the delayed germination phenotype was consistent with the previous transformants (data not shown). Despite this delay, normal yeast cell morphogenesis was evident at 37°. In addition, Δ*ystA* germination and growth in macrophages displayed no clear difference compared to the wild-type (data not shown), suggesting that host-derived signals can override the observed delay. Because *ystA* has one short translated ORF (ORF1) that is not conserved in other species and one ORF (ORF2) that is conserved in closely related species, alleles were generated that mutated the initiator ATG codon of either ORF1 [*ystA^M^*^(ORF1)L^] or ORF2 [*ystA^M^*^(ORF2)L^], and these were used to transform a Δ*ystA* strain. Phenotypic examination of these strains showed that the allele containing the ATG mutation in ORF1 failed to complement the germination delay of the Δ*ystA* strain, whereas the allele with the ATG mutation in ORF2 fully complemented this phenotype (Figure 7B).

The high conservation of ORF2, but not of the functional ORF1, among these closely related species was unexpected. To examine this further, the *ystA* region was PCR-amplified from three clinical isolates of *P. marneffei* (FRR3840, 3841, and 4059), cloned, and sequenced. DNA sequence alignment identified a single nonsynonymous base substitution difference between FRR2161/FRR3841 and FRR3840/FRR4059 over the 206 bp of ORF1. Alignment of a 206-bp region of the adjacent *bstA* gene identified a single nonsynonymous nucleotide difference between FRR2161/FRR3840/FRR4059 and FRR3841. Thus, *ystA* produces ∼850 base transcript with a long 3′ UTR and encodes a short 68 aa protein unique to *P. marneffei* that is involved in germination at 37°. The biochemical activity of YstA is currently obscure.

### Genes predominantly expressed in yeast cells include those involved in interactions with the host cell and fundamental to infection

The cell wall of fungi plays a vital role in the survival and growth of the cell. Its composition and structure have been shown to affect adhesion and virulence in a number of pathogens (Latgé 2007; Lesage and Bussey 2006; Sosinska *et al.* 2009). The cell wall typically comprises cross-linked glycans, chitins, and cell wall proteins. Chitin is a homopolymer of 1,4-N-acetylglucosamine and is found in all fungi. In *C. albicans*, four chitin synthases are responsible for the production of chitin, which has a vital role in cell shape and viability and is responsible for creating layers in the inner walls of all cell types (Munro and Gow 2001; Munro *et al.* 2003). Chitin is used to mask *C. albicans* from the host pathogen recognition receptor Dectin-1 (Mora-Montes *et al.* 2011). The chitin synthase encoded by *chsE* shows upregulated expression in *P. marneffei* yeast cells compared to hyphae. The ortholog in *P. brasiliensis* also has higher expression in yeast cells compared to hyphae, and this correlates with an increase in chitin production (Barreto *et al.* 2009).

Additionally involved in the cell wall glucan/chitin matrix is the ortholog of the glycosyl hydrolase Dfg5, which in *N. crassa* contributes to the covalent cross-linking of glycoproteins into the cell wall (Latgé 2007). Cell wall glycoproteins are structural components vital for cell wall biogenesis and the glycoprotein content of cell walls varies between particular cell types, allowing specialized adaptation to specific environmental stresses (Latgé 2007; Sosinska *et al.* 2009). In *N. crassa*, *dfg5* mutant cells have irregular hyphal diameters, exhibit dichotomous branching, secrete higher levels of cell wall proteins, and are sensitive to various stress reagents (Maddi *et al.* 2012). Finally, the ability of fungal cells to adhere to various host tissues is fundamental to successful infection. Another gene upregulated in established yeast cells encodes E1 beta subunit pyruvate dehydrogenase. Apart from its major metabolic roles in converting pyruvate into acetyl-CoA, pyruvate dehydrogenase and its complex have been associated with adhesion to host tissue, particularly to fibronectin in *Mycoplasma mycoides* and *Mycoplasma pneumonia* (Dallo *et al.* 2002; Jores *et al.* 2009).

**Figure 8:**
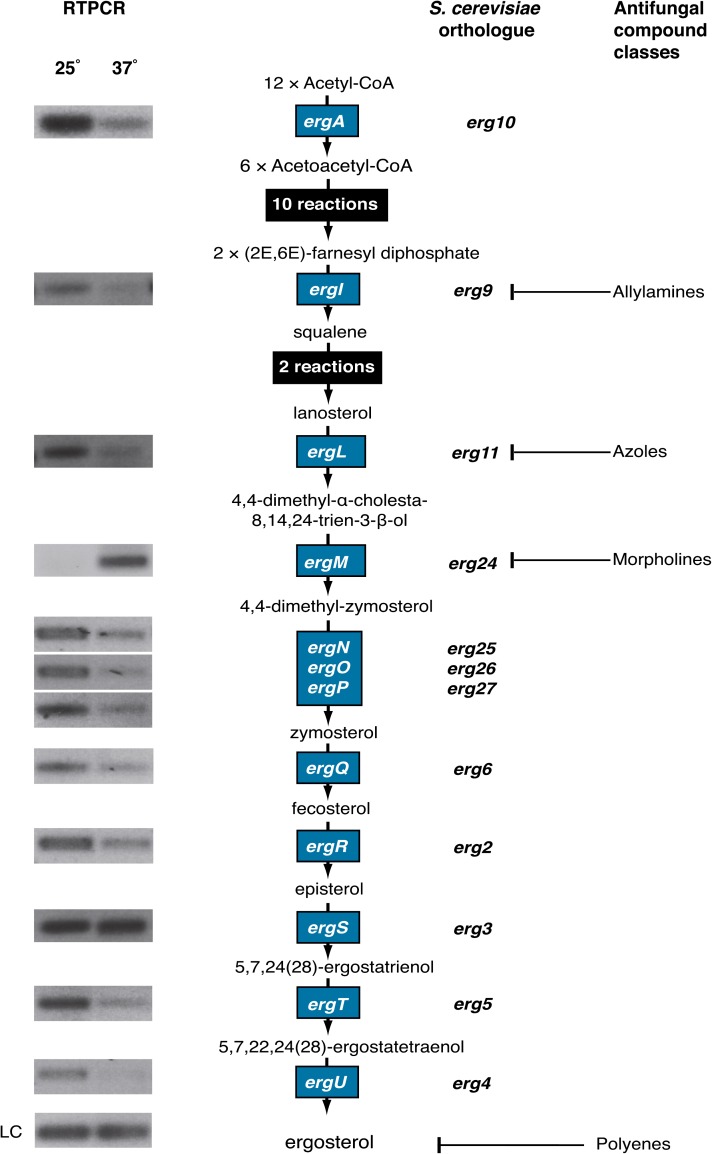
Ergosterol pathway expression analysis. The ergosterol biosynthesis pathway from acetyl-CoA to ergosterol including the chemical intermediates and genes encoding the enzymes for each step. Expression of *P. marneffei* genes indicated by blue boxes were examined by RT-PCR using RNA from vegetative hyphal cells grown at 25° for 2 d, yeast cells grown at 37° for 2 d, and primers specific for each gene. *S. cerevisiae* orthologs are shown adjacent to the *P. marneffei* genes. The proteins targeted by various classes of antifungal compound are also indicated. LC, loading control (*benA*).

Ergosterol is a key component of the fungal cell membrane, shown to control fluidity and formation of lipid rafts (Brown and London 2000; Lees *et al.* 1980; Sharma 2006). Lipid rafts allow the stable association of membrane-bound proteins to the cell surface, thus ergosterol production has been linked to many important cellular functions from nutrient uptake and drug efflux to polarity determination (Alvarez *et al.* 2007; Iwaki *et al.* 2008; Pasrija *et al.* 2008). The biochemistry and genetics involved in ergosterol biosynthesis and sterol biosynthesis more broadly is well-characterized in several species and has become the target of several antifungal compound classes (Nes 2011). The C-14 sterol reductase encoding gene *ergM*, active in the ergosterol biosynthetic pathway, showed differentially upregulated expression throughout yeast cell growth in *P. marneffei* (Figure 5 and Figure 8).

Ergosterol biosynthesis is dependent on oxygen and iron. Regulation of the ergosterol biosynthetic pathway in the variable environment of the host, where both of these may be limiting, is therefore vital (Jones 1986; Weinberg 1986). RT-PCR validation of *ergM* expression confirmed that it was upregulated at 37°. Although it was not surprising that ergosterol biosynthesis may differ between hyphal cells growing at 25° and yeast cells at 37°, to compensate for membrane fluidity differences at the two temperatures, *ergM* was the only upregulated gene in the ergosterol biosynthesis pathway (Figure 8). This suggests that the reduction of C-14 on the sterol backbone was more important for sterol function at 37° than at 25°. Alternatively, the biochemical activity of ErgM, C-14 reduction, may be the limiting step in ergosterol biosynthesis at 37° and *ergM* is upregulated to increase flux through the pathway.

**Figure 9:**
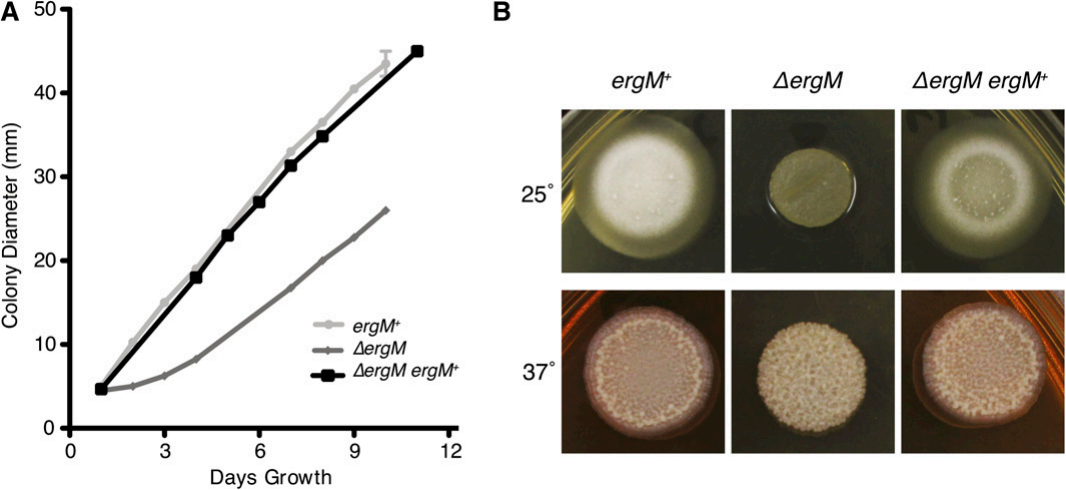
Growth rate defect of the Δ*ergM* strain. (A) Radial growth rate (colony diameter) was measured every 24 hr for the control (*ergM^+^*), deletion (Δ*ergM*), and complemented (Δ*ergM ergM^+^*) strains at 25° on ANM medium. (B) Colony morphology after 4 d of growth on BHI medium at 25° and 37°.

**Figure 10:**
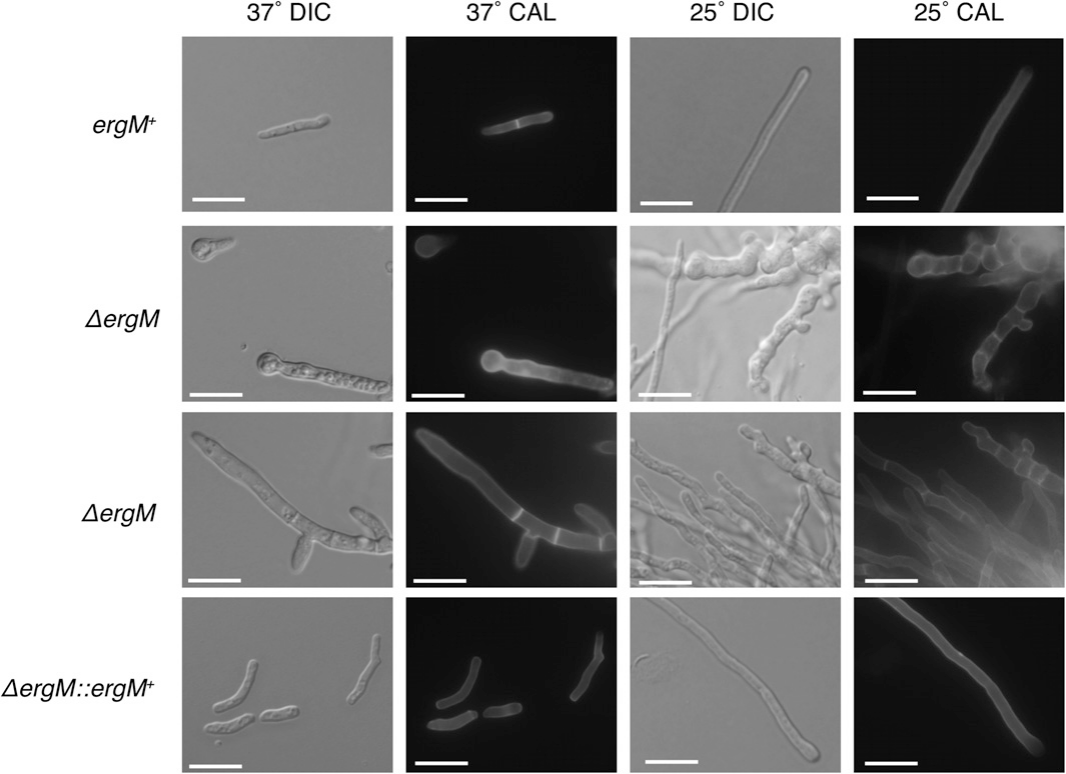
Microscopic examination of Δ*ergM* strain. Control (*ergM^+^*), deletion (*ΔergM*), and complemented (Δ*ergM ergM^+^*) strains were grown on 2% BHI for 4 d at the indicated temperature and examined at 100× magnification. DIC, differential interference contrast microscopy; calcofluor (CAL), 0.1 mg/ml calcofluor staining under UV filter; scale bars, 20 µm.

To assess the role of *ergM* during growth and morphogenesis in *P. marneffei*, the *ergM* coding sequence was deleted by homologous gene replacement (see *Materials and Methods* section). Deletion of *ergM* led to significant and pleiotropic effects, including swollen and highly branched cells, slow growth, delayed conidiation, and altered antifungal sensitivity. The most obvious of these was a severe reduction in growth rate and altered colony morphology at both 25° and 37° (Figure 9). Microscopic analysis showed normal levels of progression past the germling stage but severe polarity defects in a proportion of cells at 25° (Figure 10), whereas at 37° a large proportion of the conidia failed to produce more than a small germ tube. The remaining cells grew at a reduced rate with very few yeast cells produced (Figure 10). The more severe phenotype at 37° confirmed the importance of *ergM* at this temperature. The growth reduction of the *ergM* deletion strain at both temperatures was initially severe; however, there was a marked recovery in growth over time (Figure 10). This recovery was not attributable to the accumulation of suppressor mutations because conidia from the deletion strain exhibited the severe growth phenotype of the original transformant at both temperatures (data not shown). The phenotypic change was therefore a biochemical or regulatory response to the altered sterol production. This phenomenon might be akin to a response observed in *S. cerevisiae*, in which other membrane components such as phospholipids were altered in ergosterol biosynthesis mutants, allowing growth (Sharma 2006).

Ergosterol biosynthesis is the target of several classes of antifungal compounds, including azoles (*ergL*) and morpholines (*ergM*) (Figure 8). Amphotericin B (an allylamine) is commonly used to treat *P. marneffei* infections and also binds ergosterol itself to effect cell death. Therefore, it might be expected that deletion of an enzyme in the ergosterol biosynthesis pathway would result in resistance to these antifungals. Although resistance to amphotericin B was increased in the *ergM* deletion strain, this strain showed increased sensitivity to the azoles fluconazole and voriconazole (Table 3). This implies that either *ergM* or *ergL* was required for the production of the functional sterol because the loss of ErgL and ErgM activity had a combinatorial rather than epistatic effect on phenotype. In other species, the deletion of some ergosterol biosynthesis genes results in a modified sterol profile (Alcazar-Fuoli *et al.* 2008; Geber *et al.* 1995; Ruan *et al.* 2002). As such, deletion of biosynthetic genes does not eliminate sterol production but does modify the type of sterol produced. This modification may lead to reduced binding of amphotericin B to the sterol and could explain the resistance of the *ergM* deletion to this compound. These experiments suggest that combinatorial treatment of *P. marneffei* infections with recently developed compounds targeting *ergM* (Hata *et al.* 2010; Liang *et al.* 2009) and azoles would be effective, with a loss of ErgL and ErgM activity having a combinatorial effect on growth. Conversely, combination of compounds targeting ErgM with allylamines may not be effective because a loss of *ergM* leads to resistance to the allylamine amphotericin B.

**Table 3:** Δ*ergM* sensitivity to antifungal drugs measured as minimum inhibitory concentrations using e-test strips

	25°			37°		
	*ergM^+^*	Δ*ergM*	Δ*ergM ergM^+^*	*ergM^+^*	Δ*ergM*	Δ*ergM ergM^+^*
Voriconazole	0.032	0.001	0.032	0.006	<0.001	0.006
Fluconazole	24	0.5	24	3	0.125	3
Amphotericin B	>32	>32	>32	1.5	4	1.5

Minimum inhibitory concentration values are presented as µg/ml concentrations.

In a recent microarray study, Lin *et al.* (2012) compared the hyphal-specific and yeast-specific gene expression profiles of *P. marneffei* clinical isolate B-6323, rather than the type strain used here. Although the microarray design encompassed most annotated gene models, the study was restricted to 1884 *P. marneffei* genes orthologous to *S. cerevisiae* genes with identified GO terms. Despite the different strains and substantially different growth conditions that were used, 76% of the top 400 differentially expressed genes with respect to hyphal cells and yeast cells identified in the present study were also identified by Lin *et al.* (2012). Of these genes, 73% were consistent with respect to which cell type displayed higher gene expression. Numerous cell-type–specific gene expression differences identified in the present study are also evident in similar studies of other dimorphic fungi such as *P. brasiliensis* and *H. capsulatum*. For example, when comparing the differentially expressed genes identified in *H. capsulatum* by Hwang *et al.* (2003) and those identified in *P. marneffei* (Figure 5) (37° *vs.* 25°), 55% show similar patterns of expression between hyphal cells and yeast cells. Interestingly, of the 27% of genes that were inconsistent between this study and that of Lin *et al.* (2012) in terms of the cell type with the greater expression, 83% showed consistent cell-type expression between this study and the study by Hwang *et al.* (2003) using *H. capsulatum*. Overall, the gene expression profiles described in these studies highlight key similarities and also differences in the metabolic and reproductive modes of yeast and hyphal cells.

## Conclusions

Cell-state–specific gene expression is one of the most informative strategies for understanding the physiological properties of different types of cells in multicellular organisms. This study effectively identified genes differentially expressed in hyphal, yeast, and conidiating cultures, and those expressed early and late in hyphal and yeast cell growth of the dimorphic opportunistic pathogen *P. marneffei*. *P. marneffei* hyphal cells behave like those of many other filamentous fungi at 25°, focusing on maintaining growth through hyphal tip extensions and tightly regulated asexual reproduction. Compared to hyphal cells, the selective pressures imposed on yeast cells are very different. Survival of yeast cells in the host is dependent on their ability to survive within the host macrophage, avoiding destruction, while scavenging limited nutrients, such as iron. Genes important for high-affinity iron assimilation were upregulated throughout yeast cell growth in *P. marneffei*. A cell wall specialized for host environments is also necessary for *P. marneffei* yeast cells with possibly different chitin and cell wall protein requirements than those of hyphal cells. Similarly, the ability to adhere to the host cell is considered a pathogenicity factor in many fungi, making the increased expression of these genes in yeast cells an area of great interest. Identification and characterization of *ergM* showed that this is a gene fundamental to cell wall maintenance, nutrient uptake, and polarity determination, and loss of function results in severe growth defects in *P. marneffei* and important differences in susceptibility to antifungal agents. Other genes such as *ystA* are specific to the yeast cell state and are highly restricted taxonomically, highlighting the differences between the dimorphic fungi and possibly their evolved mechanisms for growth in a host. There is much to be discovered about the specific roles of genes identified in this study; further functional analyses are necessary. What can be elucidated is that temporal and cell-specific transcriptional differences identified in this study reflect different modes of reproduction, requirements throughout development, and changing environmental pressures throughout the life cycle of *P. marneffei*.

## Supplementary Material

Supporting Information
