# Supplementary material for: Cell-Type–Specific Transcriptional Profiles of the Dimorphic Pathogen Penicillium marneffei Reflect Distinct Reproductive, Morphological, and Environmental Demands
Source: G3 (Bethesda). 2013 Nov 1;3(11):1997–2014. doi: 10.1534/g3.113.006809 (PMC3815061; doi:10.1534/g3.113.006809)
Supplement: Supporting Information [file supp_3_11_1997__index.html]

Cell-Type–Specific Transcriptional Profiles of the Dimorphic Pathogen Penicillium marneffei Reflect Distinct Reproductive, Morphological, and Environmental Demands — Supporting Information 

# Cell-Type–Specific Transcriptional Profiles of the Dimorphic Pathogen *Penicillium marneffei* Reflect Distinct Reproductive, Morphological, and Environmental Demands

## Supporting Information for Pasricha *et al.*, 2013

**Files in this Data Supplement:**

- Supporting Information - Figures S1-S3 and Tables S1-S7 (PDF, 439 KB)
- Figure S1 - Confirmation of differential gene expression (PDF, 193 KB)
- Figure S2 - Synteny across the *ystA* region is only conserved in species closely related to *P. marneffei*. (PDF, 116 KB)
- Figure S3 - The *ystA* genomic region encodes a single transcript. (PDF, 211 KB)
- Table S1 - Oligonucleotides used in this study (.xls, 23 KB)
- Table S2 - Relative expression data for the three cell states (.xls, 2 MB)
- Table S3 - Fold changes in expression for temperature switching and terminal cell types (.xls, 2 MB)
- Table S4 - MeV phase-specific curated clusters (.xls, 51 KB)
- Table S5 - MeV early and late gene expression curated clusters (.xls, 59 KB)
- Table S6 - GO associations for phase-specific expression clusters (.xls, 172 KB)
- Table S7 - GO associations for early and late expression clusters (.xls, 203 KB)
