# Supplementary material for: Cell-Type–Specific Transcriptional Profiles of the Dimorphic Pathogen Penicillium marneffei Reflect Distinct Reproductive, Morphological, and Environmental Demands
Source: G3 (Bethesda). 2013 Nov 1;3(11):1997–2014. doi: 10.1534/g3.113.006809 (PMC3815061; doi:10.1534/g3.113.006809)
Supplement: Supporting Information [file supp_g3.113.006809_006809SI.pdf]

G3·Genes | Genomes | Genetics

Cell type specific transcriptional profiles of the dimorphic pathogen *Penicillium marneffei* reflect distinct reproductive, morphological and environmental demands.

*S. Pasricha[1], M. Payne[1], D.Canovas[1,2], L. Pase[1,3], N. Ngaosuwankul[1,4], S. Beard[1,5], A. Oshlack[6,7], G. K. Smyth[6,8], S. C. Chaiyaroj[4], K. J. Boyce[1] and A. Andrianopoulos[1] **

1. Department of Genetics, University of Melbourne, Victoria 3010, Australia
2. Present address: Departamento de Genética, Facultad de Biología, Universidad de Sevilla, 41012 Sevilla, Spain
3. Present address: Institute of Toxicology and Genetics, Karlsruhe Institute of Technology, Hermann-von-Helmholtz-Platz 1 76344 Eggenstein-Leopoldshafen, Germany
4. Department of Microbiology, Faculty of Science, Mahidol University, Rama VI Road, Bangkok 10400, Thailand.
5. Present address: Welcome Trust Centre for Cell Biology, University of Edinburgh, Edinburgh EH93JR, United Kingdom
6. Bioinformatics Division, Walter and Eliza Hall Institute of Medical Research, Parkville, Victoria, 3052
7. Present address: Murdoch Children's Research Institute, 50 Flemington Road, Parkville, Victoria 3052, Australia
8. Department of Mathematics and Statistics, University of Melbourne, Victoria 3010, Australia

* Corresponding author: Alex Andrianopoulos, Department of Genetics, University of Melbourne, Victoria 3010, Australia. Telephone + 61 3 8344 5164. Facsimile + 61 3 8344 5139. Email alex.a@unimelb.edu.au

DOI: 10.1534/g3.113.006809

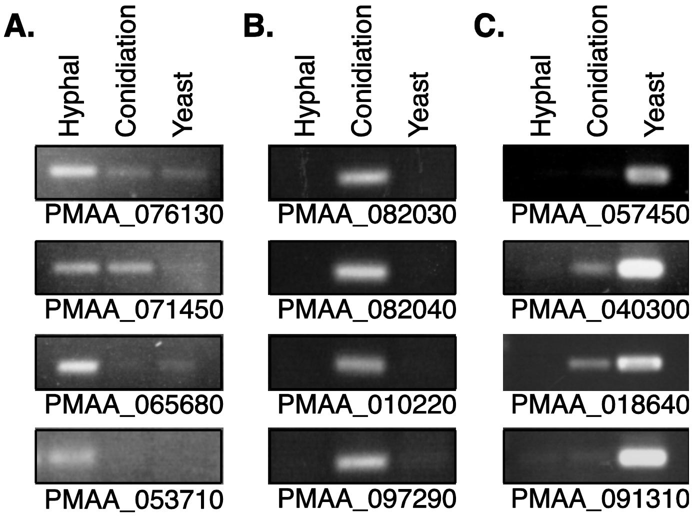

Figure S1 Confirmation of differential gene expression. The expression of a subset of differentially expressed genes identified in the microarray analysis was confirmed by RT PCR on RNA isolated from vegetative hyphae at 25° (A), asexual developing cultures at 25° (B) and from yeast cells at 37° (C). Genes shown are as follows: Hyphal specific; PMAA_076130 meiosis induction protein kinase (Ime2), PMAA_071450 hypothetical protein, PMAA_065680 nucleoside-diphosphate-sugar epimerase, PMAA_053710 hypothetical protein and PMAA_018570 allergen Asp F3. Asexual development specific; PMAA_082030 conidial pigment biosynthesis 1,3,6,8-tetrahydroxynaphthalene reductase Arp2, PMAA_082040 conidial pigment biosynthesis scytalone dehydratase Arp1, PMAA_010220 glutaminase GtaA, PMAA_075300 C2H2 type conidiation transcription factor BrlA and PMAA_097290 hypothetical protein. Yeast specific; PMAA_057450 ferrooxidoreductase Fet3, PMAA_040300 sodium P-type ATPase, PMAA_018640 cytochrome P450 monooxygenase, PMAA_031950 4-hydroxyphenylpyruvate dioxygenase and PMAA_091310 hypothetical protein.

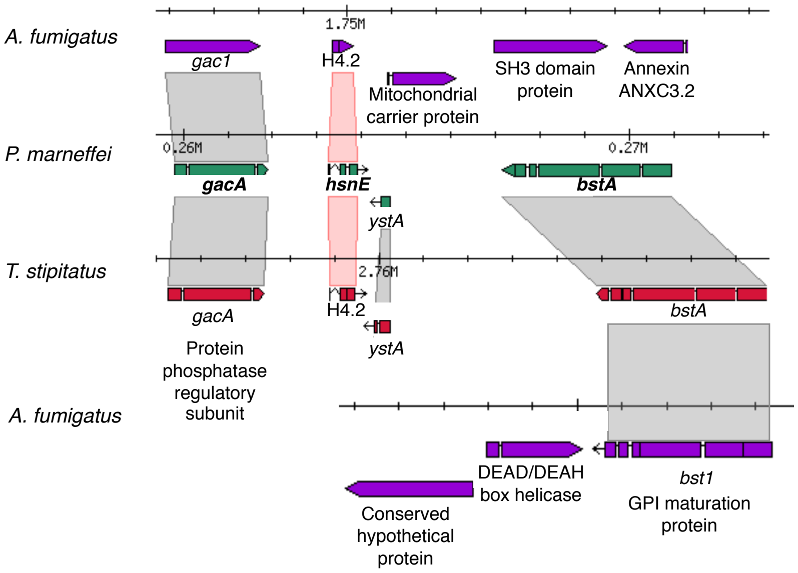

Figure S2 Synteny across the *ystA* region is only conserved in species closely related to *P. marneffei*. Diagrammatic representation of the genomic region surrounding *ystA* in *P. marneffei*, *T. stipitatus* and *A. fumigatus* showing the predicted genes and gene product. This region is syntenic in *P. marneffei* and *T. stipitatus* but disrupted in *A. fumigatus* where each side occurs on a different chromosome. There is no homology to *ystA* in *A. fumigatus*.

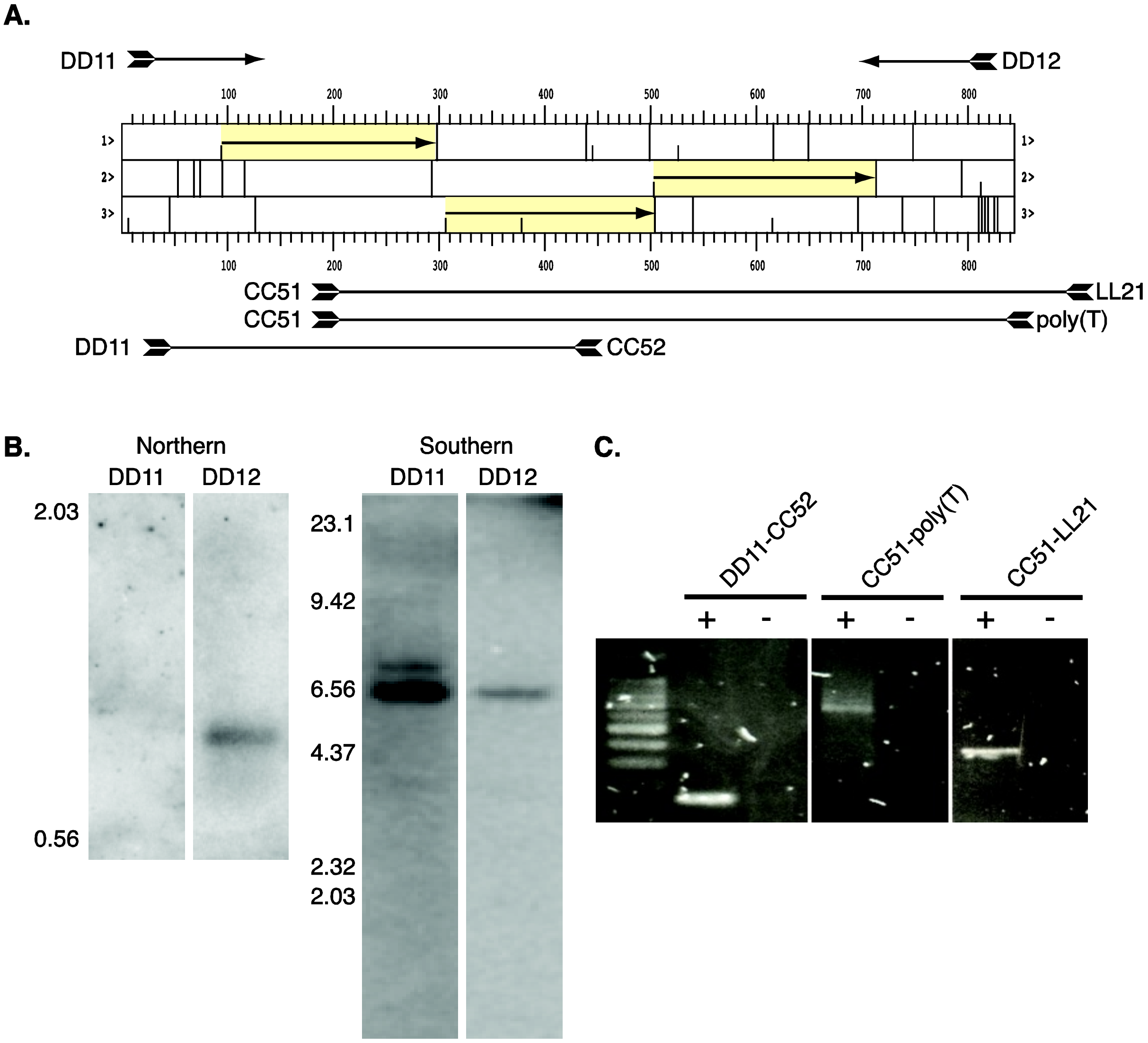

Figure S3 The *ystA* genomic region encodes a single transcript. (A) Diagrammatic representation of the three reading frames with predicted ORFs longer than 50 aa in an 850 bp region identified by the 1E11 microarray probe. The DD11 and DD12 arrows represent oligonucleotides used to generate single stranded probes of this region to identify transcripts. The shaded regions (yellow) with arrows define the three ORFs in this region that are 68, 65 and 69 aa in length. The CC51-LL21, CC51-poly(T) and DD1-CC52 oligonucleotide primer pairs were used in RT-PCR experiments to define the boundaries of the transcript in this region. (B) Single stranded radiolabelled probes were generated using DD11 and DD12 and these were used to probe a northern blot with total *P. marneffei* RNA isolated from yeast cells at 37°. A single transcript of approximately 850 bases was detected with the DD12-derived probe only. To check the quality of the probes each was tested in a Southern blot hybridisation using total genomic DNA digested with BamHI. The predicted 6.5 kb genomic DNA fragment was identified with both probes. (C) RT-PCR using the primer pairs indicated in A was performed with total RNA isolated from yeast cells grown at 37°. To assess the amount of contaminating genomic DNA in the samples both reverse transcriptase (+) and no reverse transcriptase (-) reactions were performed.

Tables S1-S7
Available for download at http://www.g3journal.org/lookup/suppl/doi:10.1534/g3.113.006809/-/DC1

Table S1 Oligonucleotides used in this study

Table S2 Relative expression data for the three cell states.

Table S3 Fold changes in expression for temperature switching and terminal cell types.

Table S4 MeV phase-specific curated clusters.

Table S5 MeV early and late gene expression curated clusters

Table S6 GO associations for phase-specific expression clusters.

Table S7 GO associations for early and late expression clusters.
