# Supplementary material for: Cell-Type–Specific Transcriptional Profiles of the Dimorphic Pathogen Penicillium marneffei Reflect Distinct Reproductive, Morphological, and Environmental Demands
Source: G3 (Bethesda). 2013 Nov 1;3(11):1997–2014. doi: 10.1534/g3.113.006809 (PMC3815061; doi:10.1534/g3.113.006809)
Supplement: Supporting Information [file supp_g3.113.006809_FigureS1.pdf]

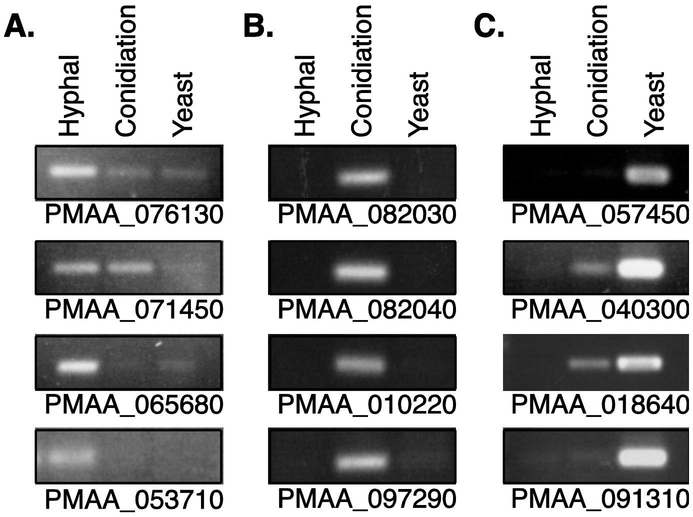

Figure S1 Confirmation of differential gene expression. The expression of a subset of differentially expressed genes identified in the microarray analysis was confirmed by RT PCR on RNA isolated from vegetative hyphae at 25° (A), asexual developing cultures at 25° (B) and from yeast cells at 37° (C). Genes shown are as follows: Hyphal specific; PMAA_076130 meiosis induction protein kinase (Ime2), PMAA_071450 hypothetical protein, PMAA_065680 nucleoside-diphosphate-sugar epimerase, PMAA_053710 hypothetical protein and PMAA_018570 allergen Asp F3. Asexual development specific; PMAA_082030 conidial pigment biosynthesis 1,3,6,8-tetrahydroxynaphthalene reductase Arp2, PMAA_082040 conidial pigment biosynthesis scytalone dehydratase Arp1, PMAA_010220 glutaminase GtaA, PMAA_075300 C2H2 type conidiation transcription factor BrlA and PMAA_097290 hypothetical protein. Yeast specific; PMAA_057450 ferrooxidoreductase Fet3, PMAA_040300 sodium P-type ATPase, PMAA_018640 cytochrome P450 monooxygenase, PMAA_031950 4-hydroxyphenylpyruvate dioxygenase and PMAA_091310 hypothetical protein.
