# Supplementary material for: Cell-Type–Specific Transcriptional Profiles of the Dimorphic Pathogen Penicillium marneffei Reflect Distinct Reproductive, Morphological, and Environmental Demands
Source: G3 (Bethesda). 2013 Nov 1;3(11):1997–2014. doi: 10.1534/g3.113.006809 (PMC3815061; doi:10.1534/g3.113.006809)
Supplement: Supporting Information [file supp_g3.113.006809_FigureS2.pdf]

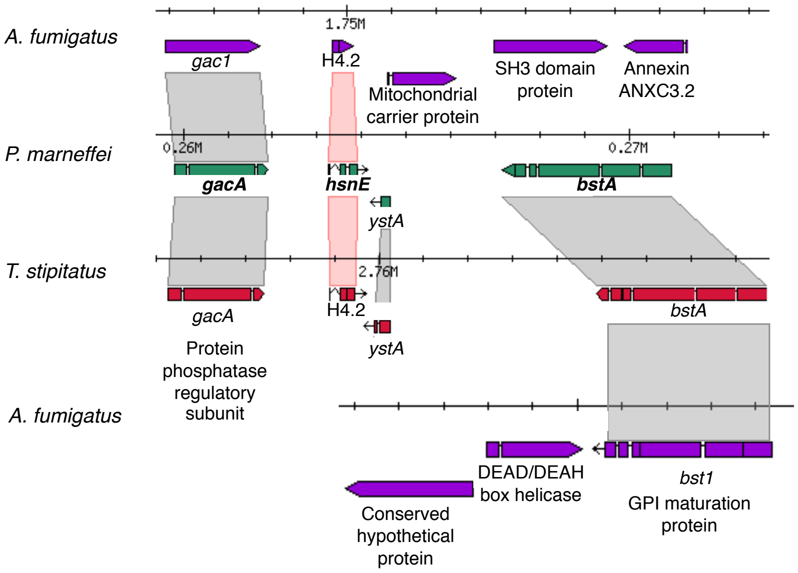

Figure S2 Synteny across the *ystA* region is only conserved in species closely related to *P. marneffei*. Diagrammatic representation of the genomic region surrounding *ystA* in *P. marneffei*, *T. stipitatus* and *A. fumigatus* showing the predicted genes and gene product. This region is syntenic in *P. marneffei* and *T. stipitatus* but disrupted in *A. fumigatus* where each side occurs on a different chromosome. There is no homology to *ystA* in *A. fumigatus*.
