# Supplementary material for: Cell-Type–Specific Transcriptional Profiles of the Dimorphic Pathogen Penicillium marneffei Reflect Distinct Reproductive, Morphological, and Environmental Demands
Source: G3 (Bethesda). 2013 Nov 1;3(11):1997–2014. doi: 10.1534/g3.113.006809 (PMC3815061; doi:10.1534/g3.113.006809)
Supplement: Supporting Information [file supp_g3.113.006809_FigureS3.pdf]

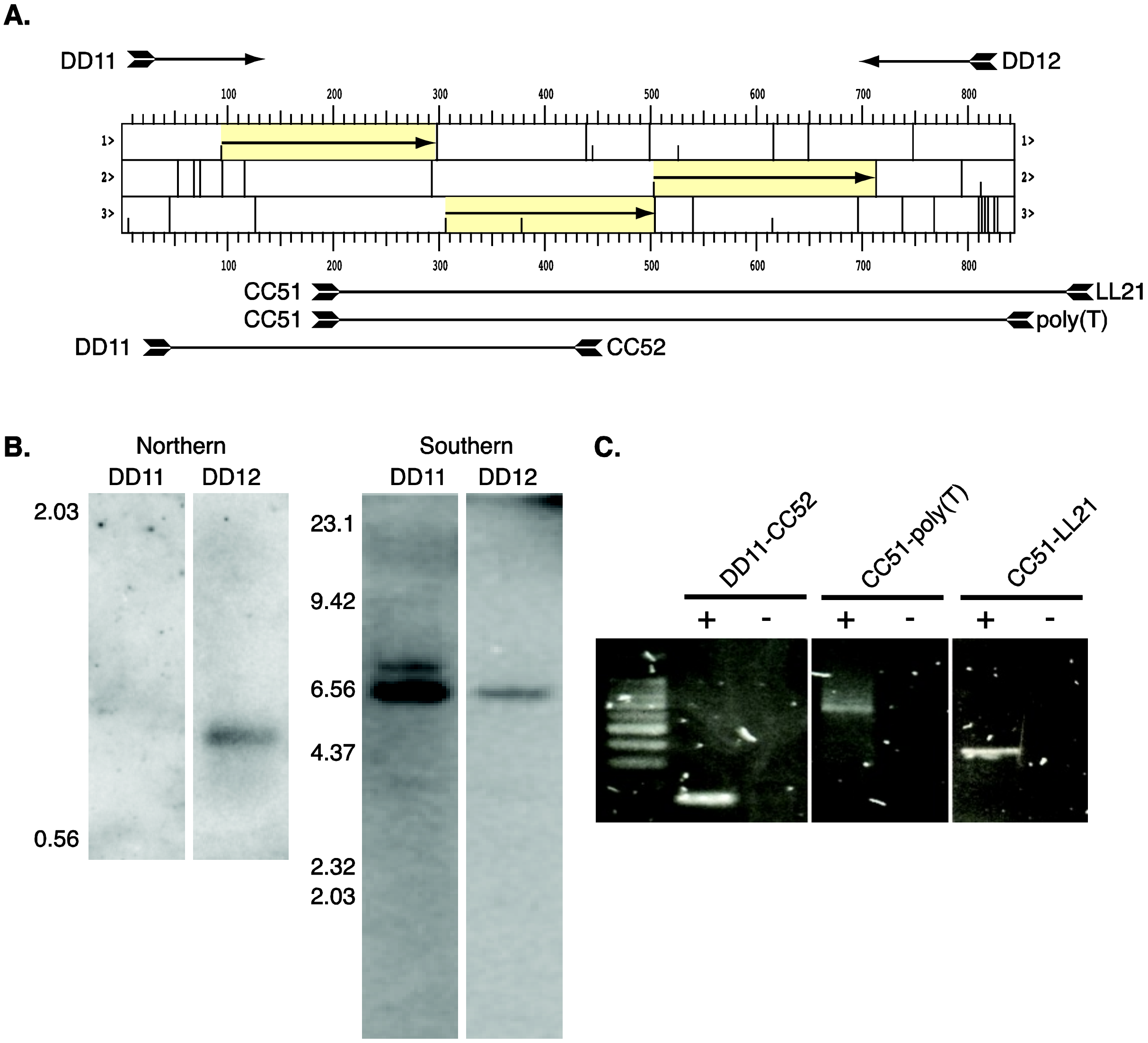

Figure S3 The *ystA* genomic region encodes a single transcript. (A) Diagrammatic representation of the three reading frames with predicted ORFs longer than 50 aa in an 850 bp region identified by the 1E11 microarray probe. The DD11 and DD12 arrows represent oligonucleotides used to generate single stranded probes of this region to identify transcripts. The shaded regions (yellow) with arrows define the three ORFs in this region that are 68, 65 and 69 aa in length. The CC51-LL21, CC51-poly(T) and DD1-CC52 oligonucleotide primer pairs were used in RT-PCR experiments to define the boundaries of the transcript in this region. (B) Single stranded radiolabelled probes were generated using DD11 and DD12 and these were used to probe a northern blot with total *P. marneffei* RNA isolated from yeast cells at 37°. A single transcript of approximately 850 bases was detected with the DD12-derived probe only. To check the quality of the probes each was tested in a Southern blot hybridisation using total genomic DNA digested with BamHI. The predicted 6.5 kb genomic DNA fragment was identified with both probes. (C) RT-PCR using the primer pairs indicated in A was performed with total RNA isolated from yeast cells grown at 37°. To assess the amount of contaminating genomic DNA in the samples both reverse transcriptase (+) and no reverse transcriptase (-) reactions were performed.
